# Spatial transcriptomics reveal basal sex differences in supraoptic nucleus gene expression of adult rats related to cell signaling and ribosomal pathways

**DOI:** 10.1186/s13293-023-00554-3

**Published:** 2023-10-19

**Authors:** Dianna H. Nguyen, Victor Duque, Nicole Phillips, André Souza Mecawi, J. Thomas Cunningham

**Affiliations:** 1 Department of Physiology and Anatomy, School of Biomedical Sciences, UNT Health Science Center, Fort Worth, TX USA; 2 Texas College of Osteopathic Medicine, UNT Health Science Center, Fort Worth, TX USA; 3 Department of Biophysics, Laboratory of Molecular Neuroendocrinology, Paulista School of Medicine, Federal University of São Paulo, São Paulo, Brazil; 4 Department of Microbiology, Immunology, and Genetics, School of Biomedical Sciences, UNT Health Science Center, Fort Worth, TX USA

**Keywords:** Sex differences, Spatial transcriptomics, Supraoptic nucleus

## Abstract

**Background:**

The supraoptic nucleus (SON) of the hypothalamus contains magnocellular neurosecretory cells that secrete the hormones vasopressin and oxytocin. Sex differences in SON gene expression have been relatively unexplored. Our study used spatially resolved transcriptomics to visualize gene expression profiles in the SON of adult male (*n* = 4) and female (*n* = 4) Sprague-Dawley rats using Visium Spatial Gene Expression (10x Genomics).

**Methods:**

Briefly, 10-μm coronal sections (~ 4 × 4 mm) containing the SON were collected from each rat and processed using Visium slides and recommended protocols. Data were analyzed using 10x Genomics’ Space Ranger and Loupe Browser applications and other bioinformatic tools. Two unique differential expression (DE) analysis methods, Loupe Browser and DESeq2, were used.

**Results:**

Loupe Browser DE analysis of the SON identified 116 significant differentially expressed genes (DEGs) common to both sexes (e.g., *Avp* and *Oxt*), 31 significant DEGs unique to the males, and 73 significant DEGs unique to the females. DESeq2 analysis revealed 183 significant DEGs between the two groups. Gene Ontology (GO) enrichment and pathway analyses using significant genes identified via Loupe Browser revealed GO terms and pathways related to (1) neurohypophyseal hormone activity, regulation of peptide hormone secretion, and regulation of ion transport for the significant genes common to both males and females, (2) G_i_ signaling/G-protein mediated events for the significant genes unique to males, and (3) potassium ion transport/voltage-gated potassium channels for the significant genes unique to females, as some examples. GO/pathway analyses using significant genes identified via DESeq2 comparing female vs. male groups revealed GO terms/pathways related to ribosomal structure/function. Ingenuity Pathway Analysis (IPA) identified additional sex differences in canonical pathways (e.g., ‘Mitochondrial Dysfunction’, ‘Oxidative Phosphorylation’) and upstream regulators (e.g., CSF3, NFKB complex, TNF, GRIN3A).

**Conclusion:**

There was little overlap in the IPA results for the two different DE methods. These results suggest sex differences in SON gene expression that are associated with cell signaling and ribosomal pathways.

**Supplementary Information:**

The online version contains supplementary material available at 10.1186/s13293-023-00554-3.

## Highlights

- Successful differentiation of nuclei from myelinated fiber tracts by gene cluster analysis and identification of SON genes that are sex-specific and common to both sexes using spatial transcriptomics.
- Two differential expression analysis methods: Loupe Browser and DESeq2, generated distinct lists of differentially expressed genes for the SON.
- GO enrichment/pathway analyses reveal terms common to both sexes relevant to SON function.
- Data suggest sex differences in SON gene expression may be related to cell signaling and ribosomal pathways, possibly associated with differences in upstream regulators.

**Supplementary Information:**

The online version contains supplementary material available at 10.1186/s13293-023-00554-3.

## Abstract (plain language summary)

The brain releases the hormones oxytocin and vasopressin from the supraoptic nucleus. Oxytocin is involved in maternal behaviors, lactation, and childbirth. Vasopressin is involved in sex-based differences in social behavior and body fluid regulation. However, how the brain contributes to sex-based differences in vasopressin and oxytocin release is poorly understood. This study aimed to address this knowledge gap using spatial transcriptomics to test for sex-based differences in gene expression in the supraoptic nucleus. Spatial transcriptomics combines anatomy with gene sequencing technology, allowing us to identify groups of genes that are expressed in specific locations in the brain. We applied this approach to brain sections containing the supraoptic nucleus from four adult male and four adult female rats. Using a data analysis workflow specifically for spatial transcriptomics, we identified genes that are significantly expressed in the supraoptic nuclei of both males and females (116 genes), primarily males (31 genes), and primarily females (73 genes). Genes enriched in the supraoptic nucleus of both males and females are related to the synthesis and release of peptides like vasopressin and oxytocin. Genes specific to the male supraoptic nucleus are broadly related to cell signaling, while the female-specific genes are related to ion transporters/channels. Results from a more traditional data analysis workflow identified sex-based differences in the expression of genes related to cell metabolism and protein synthesis. Together these results may provide a mechanistic foundation that can be used to better understand how differences in gene expression related to biological sex influence brain function.

**Supplementary Information:**

The online version contains supplementary material available at 10.1186/s13293-023-00554-3.

## Background

Magnocellular neurosecretory cells (MNCs) that project to the posterior pituitary are located primarily in the supraoptic nucleus (SON) and paraventricular nucleus of the hypothalamus and in accessory cell groups in the lateral hypothalamus [1–3]. The MNCs produce two peptide hormones, oxytocin (OXT) and arginine vasopressin (AVP), that are derived from different prohormones during transport to the axon terminals in the pituitary [2]. Although the transcripts for AVP and OXT can be expressed in the same MNC, early single cell RT-PCR experiments of the SON demonstrated that most MNCs express one transcript at magnitudes higher than the other [4–6]. While some MNCs show comparable expression of both AVP and OXT, it is estimated that this only occurs in 3% of MNCs [4–6]. Therefore, most MNCs are considered to be either AVP or OXT. Action potentials, produced by depolarization of the MNCs in the hypothalamus, are propagated to the axons in the posterior pituitary triggering exocytosis and peptide release [1, 2, 7].

Changes in plasma osmolality, typically associated with dehydration, are a major determinant of circulating AVP [2, 7]. Putative AVP neurons are characterized by phasic spontaneous activity that is increased by an increase in osmolality and reduced when osmolality decreases [2, 7]. Oxytocin, acting as a circulating hormone, facilitates the milk ejection reflex and parturition [2]. Electrophysiological studies in anesthetized animals have shown that putative OXT neurons have continuous or intermittent patterns of spontaneous activity that dramatically increase before the milk ejection reflex [8]. Studies have shown that OXT neurons demonstrate changes in function to support lactation and parturition [8–10]. More recently both AVP and OXT have been reported to modulate social behavior [11, 12], although this may not be universally accepted [13]. The homogeneity of the SON has been leveraged to study neuroendocrine function, activity-dependent plasticity, and more recently transcriptomics related to the physiology of the system.

Studies of the SON transcriptome have focused on the regulation of AVP and OXT gene expression associated with dehydration and have primarily used male rats [2, 14–20]. Further, most of these studies used bulk microarray analysis and/or bulk RNAseq from punch SON samples so the transcriptomics results sacrificed spatial context. Studies on sex differences in SON gene expression are also limited. A series of studies used a microarray approach to analyze the effects of dehydration on the SONs of male and female rats [21, 22] and lactation on female rats [23]. Using a comparative transcriptomics approach, three genes, *Giot1, Tnfaip6,* and *Azin1,* were upregulated in the SON of male rats after dehydration. While these genes were difficult to detect in samples from untreated females, they were all increased in females following dehydration and by lactation. In the same study, a follow-up analysis using in situ hybridization subsequently validated the upregulation of *Giot1* and *Tnfaip2* in the SONs of lactating females and dehydrated males and females. In contrast, in situ hybridization for *Azin1* validated its increase only in dehydrated males. A Gene Ontology (GO) analysis yielded terms related to translation and cellular structure. It was concluded that the enrichment of these terms could be related to cellular adaptations to dehydration and lactation.

More recently, bulk RNAseq has been compared with microarray data sets to identify novel genes and functional pathways important to osmoregulation in the SON [19]. However, as discussed by the authors, these studies were limited by the use of punch samples that lack spatial information. The current study aims to close some of these knowledge gaps and limitations mentioned above by using the novel spatial transcriptomics technique to obtain whole transcriptomic data from the SON of adult male and female rats to investigate the basal sex differences in SON gene expression.

## Methods

### Animals

All experiment protocols involving animals were approved by the UNT Health Science Center Institutional Animal Care and Use Committee and were conducted in accordance with the National Institutes of Health *Guide for the Care and Use of Laboratory Animals*. Adult male (*n* = 4, 3–4 months, 450–600 g) and female (*n* = 4, 3–4 months, 250–400 g) Sprague–Dawley rats (Charles River Laboratories, Inc., Wilmington, MA, USA) were used in this study. Animals were housed in a temperature (24–26 °C)- and humidity (40–60%)-controlled environment with a 12-h light/dark cycle. Ad libitum access to food and water was available to the animals. Vaginal cytology was performed before euthanasia to determine the stage in the estrous cycle of the female rats. Vaginal smears were collected and evaluated as described in [24].

### Spatial transcriptomics

#### Sample preparation and imaging

**Fig. 1:**
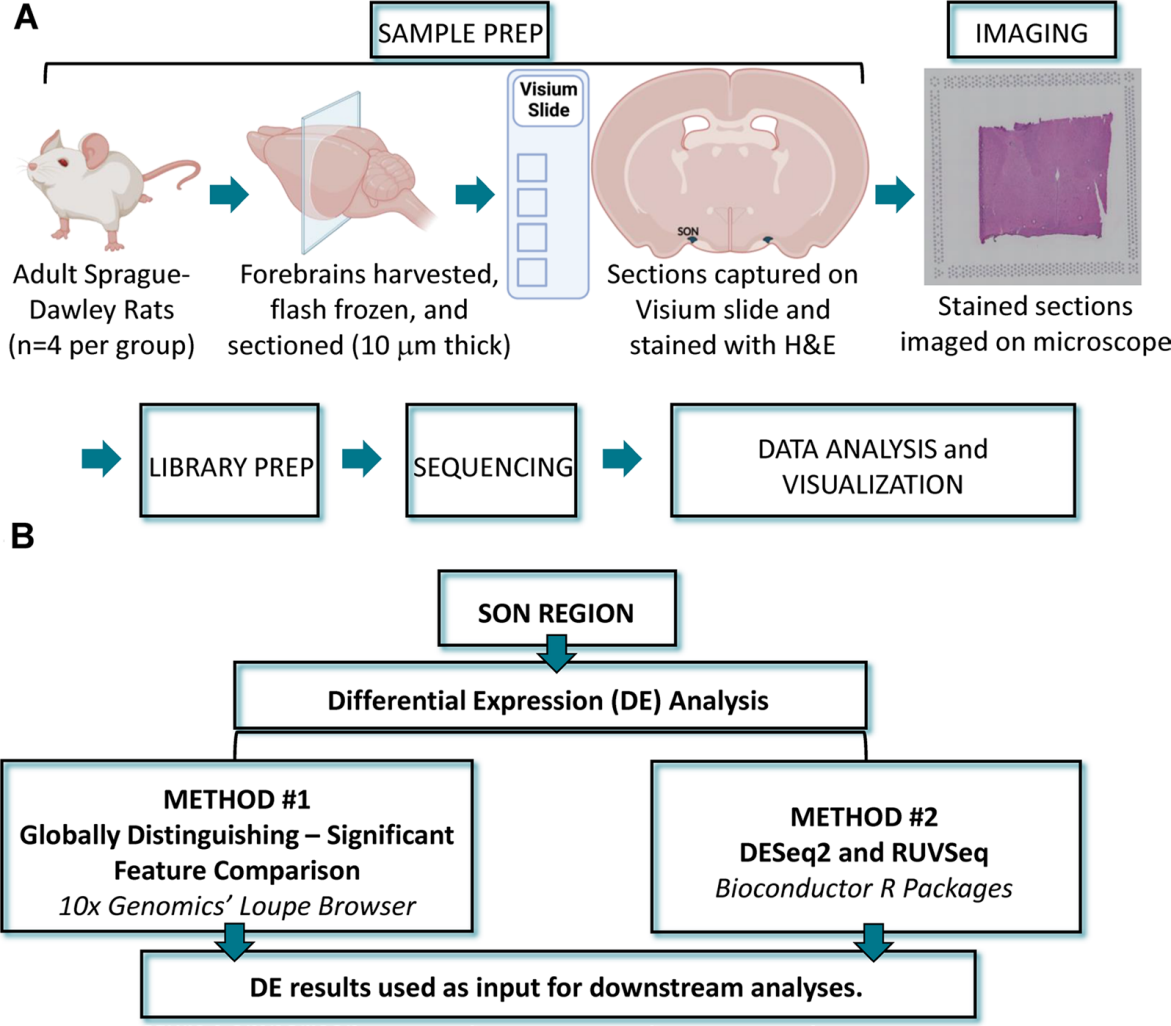
Illustrated experimental protocol and data analysis workflow. **A** Overview of experimental workflow: (1) sample preparation and imaging, (2) library preparation, (3) sequencing, and (4) data analysis/visualization. **B** Breakdown of data analysis workflow for differential expression analysis using two methods: (1) globally distinguishing—significant features comparison from 10x Genomics’ Loupe Browser and (2) DESeq2 and RUVSeq Bioconductor R packages, along with subsequent gene ontology and pathway analyses. This figure contains elements created with BioRender.com

Fresh frozen brain samples were prepared for the Visium Spatial Gene Expression (10x Genomics, Pleasanton, CA, USA) according to the manufacturer’s recommended workflow (Fig. 1A). Briefly, animals were anesthetized (2.0% isoflurane in 95% O_2_) before euthanasia by decapitation. Forebrains were harvested, flash frozen, and cut into 10-µm coronal sections. The sections were thaw-mounted onto a capture area on a Visium Spatial Gene Expression slide (PN-2000233, 10x Genomics). Each slide had four capture areas that contained approximately 5000 barcoded spots lined with spatially-barcoded probes to capture the mRNA from the samples. Each capture area was demarcated by a unique fiducial frame. The tissue was fixed and stained with hematoxylin and eosin (H&E) as described in the 10x Genomics protocol (CG000160, 10x Genomics). Brightfield images of the hematoxylin and eosin staining were captured with a 10X objective using a Keyence BZ-X810 microscope and accompanying software (BZ-X800 Viewer version 01.02.03.02 and BZ-X800 Analyzer version 1.1.2.4, Keyence Corp., Itasca, IL). The images were combined to create a fiduciary, composite image for each capture area.

#### Library construction and sequencing

After the tissue was permeabilized according to the manufacturer’s instructions, libraries were prepared and constructed according to the Visium Spatial Gene Expression User Guide (CG000239, 10x Genomics). Sequencing was performed using the NovaSeq 6000 System (Illumina, Inc., San Diego, CA, USA) at a minimum sequencing depth of 50,000 read pairs per barcode region using the following read code protocol: read 1–28 cycles, i7 index–10 cycles, i5 index–10 cycles, and read 2–90 cycles.

### Data analysis

#### Raw data processing

For each sample, corresponding raw FASTQ files and brightfield images were processed using 10x Genomics’ Space Ranger (version 1.3.0) software. The reference genome used for these analyses was Rattus norvegicus (genome assembly mRatBN7.2), which is available from the National Library of Medicine (https://www.ncbi.nlm.nih.gov/assembly/GCF_015227675.2/). Visium data were visualized and analyzed using Loupe Browser (version 6.0.0, 10x Genomics), which applied the transcriptomics data from each sample to the corresponding digital images of the coronal brain sections. In each section, one SON was identified using the composited H&E image for reference, and the associated barcoded spots were selected to be used in subsequent differential expression (DE) analyses. Please see Additional file 1 for a complete listing of all supplementary materials including figures and data files. Images of SONs identified from each sample are available in the Additional file 2: Figs. S2-9.

#### 10 × Genomics Loupe Browser

**Fig. 2:**
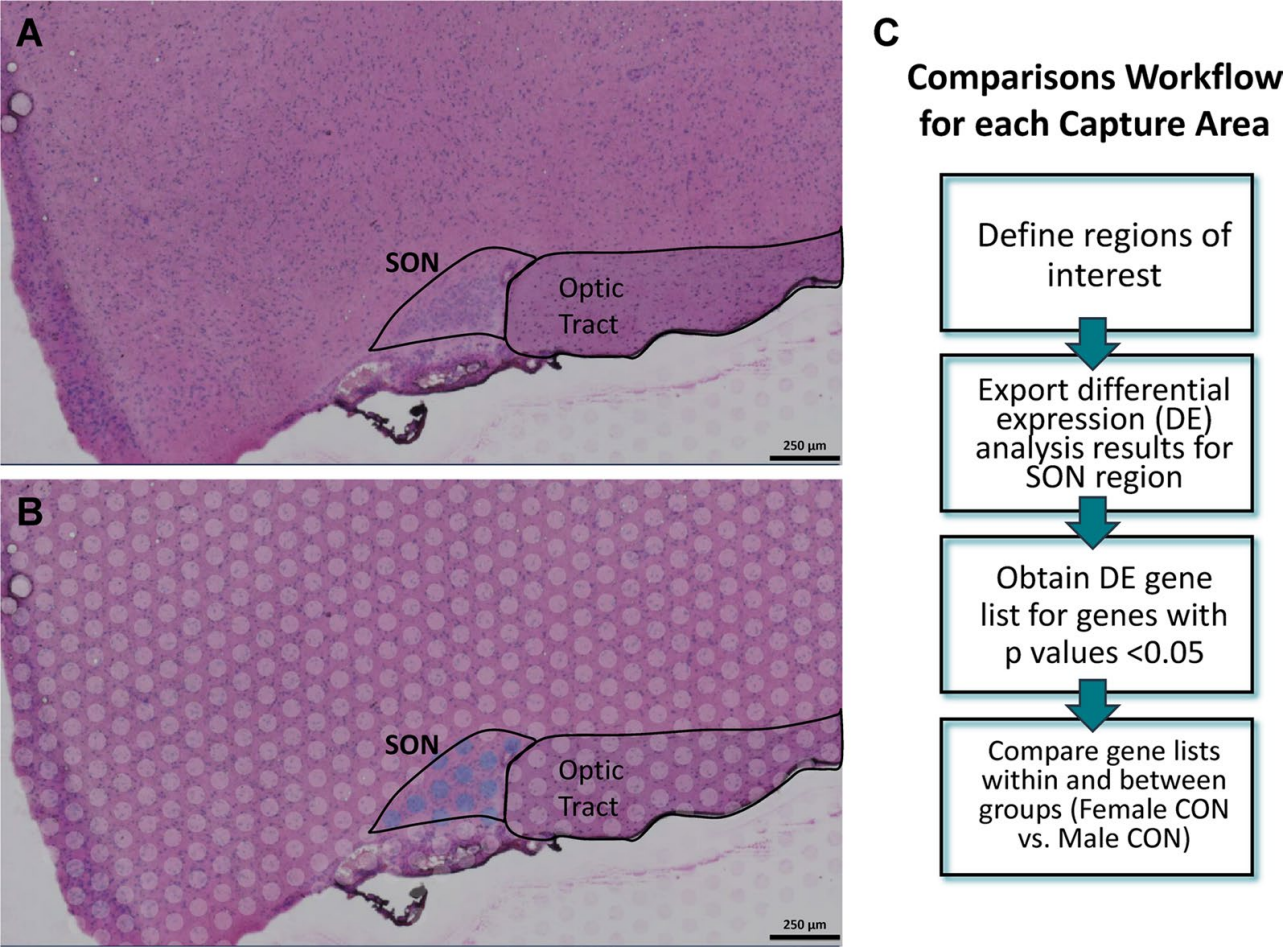
Digital images depicting supraoptic nucleus region selection and diagrammatic representation of Loupe Browser workflow. **A** Histological H&E composite images of each sample were used to locate the SON. **B** Barcoded spots overlaying the SON were then selected to define the region of interest. Images of all of the regions of interest are available in the Additional file 2: Figs. S2-9. **C** Comparison workflow for DE results generated by 10x Genomics Loupe Browser globally distinguishing method of the Significant Feature Comparison Analysis

Differentially expressed genes (DEGs) were identified using the globally distinguishing method of the Significant Feature Comparison Analysis in Loupe Browser (Fig. 1B). This analysis option compares data associated with barcoded spots from the selected cluster(s) or defined region(s) (i.e., the SON in this study) with the rest of the data set, which in our case is all barcoded spots excluding the defined SON region in the tissue section (Fig. 2A, B). This resulted in a list of significant DEGs and gene expression values (e.g., median-normalized average counts, Log2 fold change, *p*-values, etc.) for the SON. These results were exported for each sample and compiled for the two groups for downstream analyses. Currently, Loupe Browser analysis is limited to individual samples/capture areas and is unable to make comparisons between samples within the same slide or on different slides. Thus, we developed the Loupe Browser DE analysis workflow summarized in Fig. 2C to address this limitation. Significant DEGs were defined as *p*-adjusted values < 0.05 using the Benjamini–Hochberg correction for multiple tests. The significant DEGs from each capture area were compiled per group (male, *n* = 4; female, *n* = 4). Only DEGs present in all four samples per group were included in the next stage of analysis. The lists of significant DEGs and corresponding expression values for the female and male groups were sorted into three separate sets: DEGs common in both male and female groups, DEGs unique to the females, and DEGs unique to the males. Of note, Loupe Browser Significant Feature Comparison has the option of filtering the DE results to either keep (less conservative approach) or exclude (conservative approach) genes with low average counts. With the conservative approach, only genes with an average occurrence > 1 count per barcoded spot across the entire data set would be considered in the results. Analyses used DEGs determined by the less conservative approach, which is more comparable to a common DE analysis, DESeq2, discussed in later sections. Data obtained using the method in which genes with low average counts are excluded (conservative approach) are available in the Additional files 10, 11, 12, 13. In addition to DE analysis, Loupe Browser also allows for the visualization of gene cluster analysis results from Space Ranger, as well as perform spatial gene expression analysis on genes of interest. The results from the gene expression analysis can be visualized as a color-coded spatial map based on gene expression values. Highly expressing barcoded spots can be identified by setting specific threshold for the expression values. For the spatial gene expression analysis for our data set, expression values were determined for *Avp* and *Oxt*, using a log2-transformed rendering of Unique Molecular Identifier (UMI) counts that represent the absolute number of observed transcripts. The threshold used was ≥ 12.5 to ≥ 13.0 for highly expressing barcoded spots.

#### DESeq2 R analysis

A more traditional DE analysis, DESeq2 [25] which is part of the Bioconductor package, was included as a second DE method to more directly compare DEGs among the females and males (Fig. 1B). It takes count data and tests for DE based on a negative binomial distribution model. Since the samples were on different Visium slides, we controlled for the unwanted variable, a slide effect, using another Bioconductor R package, RUVSeq [26]. Barcode information from the defined SON region of each sample was exported from Loupe Browser in order to subset the count data in R, using custom scripts and functions from the Seurat package [27–30], for each sample. These data were uploaded to the Galaxy web platform, and we used the public server at usegalaxy.org [31–33] to perform the DESeq2 and RUVSeq analyses. Significant DEGs from the DESeq2 method were defined as *p*-value < 0.05. The list of DEGs and corresponding gene expression values determined by DESeq2 were used for downstream analyses. Heatmap was created using the pheatmap package in R (https://www.rdocumentation.org/packages/pheatmap/versions/1.0.12/topics/pheatmap).

#### Transcriptomics correlations

To determine how the conservative and less conservative Loupe Browse analyses output correlates with the DESeq2 outcome, we performed a Spearman correlation analysis between the Loupe Browse gene count average with the DESeq2 normalized count average, considering all males and females for both cases. Next, using published micro-punched SON bulk RNAseq [19] and Proteome [34] data, we also performed Spearman correlation analyses to compare the Visium-identified basal male rat SON genes with genes and proteins previously identified in the male rat SON.

#### Gene Ontology and pathway analyses

Gene Ontology (GO) enrichment and pathway analyses were performed on the significant DEGs lists from both DE methods using ShinyGO 0.77 [35] and cross-checking with other GO bioinformatic tools (e.g., GeneOntology, http://geneontology.org/) [35–38]. GO enrichment and pathway analysis results were visualized/summarized using R custom scripts and packages. In addition to the analyses conducted via ShinyGO, QIAGEN Ingenuity Pathway Analysis (IPA) [39] was performed to interrogate the data sets for canonical pathways, upstream regulators, and causal networks. IPA canonical pathways are characterized metabolic/cell signaling pathways based on the existing literature that are generated prior to data input. The results do not structurally change based on the data input of the study. For upstream regulators, IPA predicts which molecules are activated or inhibited to explain upregulated/downregulated genes in a given data set. Causal network analysis expands on the upstream regulator analysis by including regulators not directly connected to targets provided in a given data set. The data were analyzed and networks were generated using QIAGEN Ingenuity Pathway Analysis (IPA) (QIAGEN Inc., https://digitalinsights.qiagen.com/IPA).

#### Functional classification

To gain insights about the possible function of the significant SON DEGs unique to males, unique to females, common to both sexes, and when comparing females to males, the data obtained using the Loupe Browser and DESeq2 were mined to generate comprehensive catalogs according to its physiological and pharmacological function (IUPHAR: endogenous peptides, G protein-coupled receptors, catalytic receptors, enzymes, channels, transporters, and other pharmacological targets) [40] or as its action as transcription factors [41].

## Results

### Visium spatial transcriptomics in the SON

Using Visium, we were able to obtain the whole transcriptome results for the SON of adult male and female rats. Three of the four females were at the proestrus stage, and one was at the metestrus/diestrus stage of the estrous cycle at euthanasia.

Preliminary gene cluster analysis of the entire sections demonstrated differentiation between regions of nuclei from myelinated fiber tracts (Additional file 2: Fig. S1). For the SON, the number of barcoded spots located within each SON was around 10–12 in both groups (Additional file 2: Figs. S2–S9) with males averaging 10.75 spots and females averaging 11.25 spots, from which data obtained contributed to the results discussed subsequently. Spatial gene expression analysis in Loupe Browser targeting *Avp* and *Oxt* revealed that in both female and male rats, barcoded spots highly expressing *Avp*, *Oxt*, or both had an anatomic distribution consistent with the literature [4, 5]. The predominantly *Avp*-expressing neurons were located more in the ventral SON, *Oxt*-expressing neurons were located more in the dorsal SON, and a smaller population highly expressing both *Avp* and *Oxt* were in the region between the two distinct neuronal population (Additional file 2: Figs. S10–S17).

**Fig. 3:**
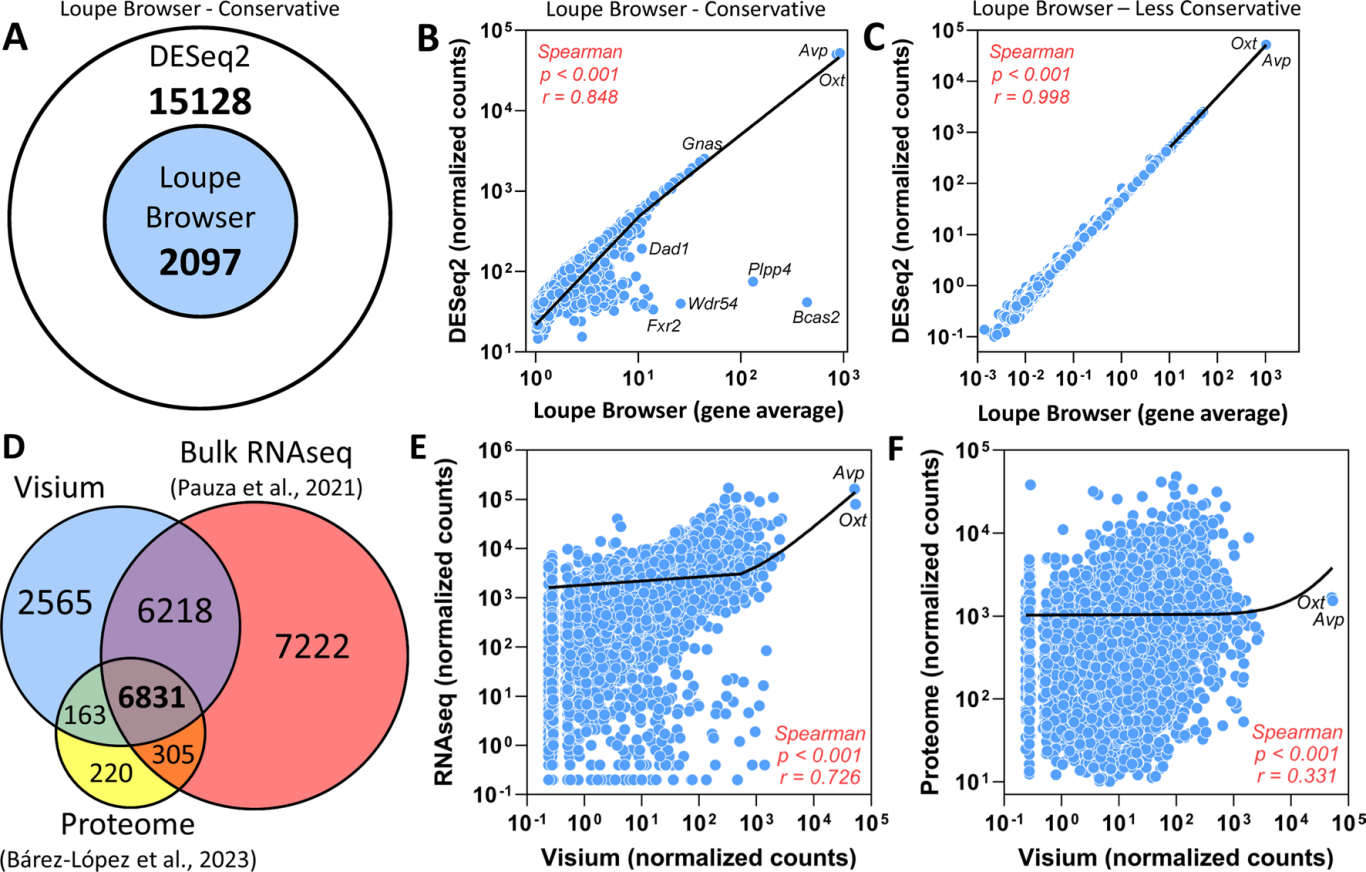
SON gene expression comparisons. **A** Euler diagram demonstrating that the 2097 genes identified by the Loupe Browser (conservative approach, genes with low average counts excluded) in the SON of male and/or female rats are among the 17,225 genes identified by DESeq2 in our Visium samples (males, *n* = 4; females, *n* = 4). **B** Data demonstrate a robust positive correlation between Loupe Browser (conservative approach) gene average and DESeq2 normalized counts in the SON for 2097 commonly discovered RNAs. **C** Data demonstrate a robust positive correlation between Loupe Browser (less conservative approach, genes with low average counts included) gene average and DESeq2 normalized counts in the SON show 100% of common genes identified by both methods. **D** Venn diagram demonstrating the overlapping of SON transcripts discovered by Visium in male rats in comparison with the previously published bulk SON RNAseq [19] and bulk SON proteome [48] data in control male rats. **E** Positive correlation between Visium and bulk SON RNAseq [19] data in control male rats. **F** Positive correlation between Visium and bulk SON proteome [48] data in control male rats

Out of ~ 34,000 genes from the mRatBN7.2 reference genome, approximately 21,000–22,000 genes were identified via Loupe Browser (Additional files 3, 4) and 17,225 genes were identified via DESeq2 (Additional file 5) in the SON. The results from these approaches were significantly correlated. All of the 2097 genes identified in the SON of male and/or female rats using the Loupe Browser conservative approach were among the 17,225 genes identified via DESeq2 (Fig. 3A) and this relationship was statistically significant (Spearman *r* = 0.848, *p* < 0.001; Fig. 3B). Similarly, all 17,225 genes identified using the DESeq2 method were also identified using the “less conservative” Loupe Browser approach. The relationship between the gene averages from Loupe Browser’s “less conservative” approach and the DESeq2 normalized counts in the SON was also statistically significant with an R-value that approached 1 (Spearman, *r* = 0.998, *p* < 0.001; Fig. 3C). Based on these results, data from the Loupe Browser “less conservative” approach was used in subsequent analyses.

In addition to comparing the number of genes identified by both DE analysis methods, we compared the Visium spatial transcriptomics data to previously published SON transcriptomic and proteomic data sets. The relationships among the spatial transcriptomics data from the male rats (*n* = 4) and previously published SON bulk RNAseq [19] and Proteome [34] data from normal male rats are illustrated in Fig. 3D. Significant positive correlations were observed for the relationships between Visium SON transcriptomics data and bulk SON RNAseq data (Spearman *r* = 0.726, *p* < 0.001; Fig. 3E) and between Visium SON transcriptomics data and bulk SON proteome data (Spearman *r* = 0.331, *p* < 0.001; Fig. 3F).

### Significant SON differentially expressed genes

#### Significant SON DEGs identified using Loupe Browser

**Fig. 4:**
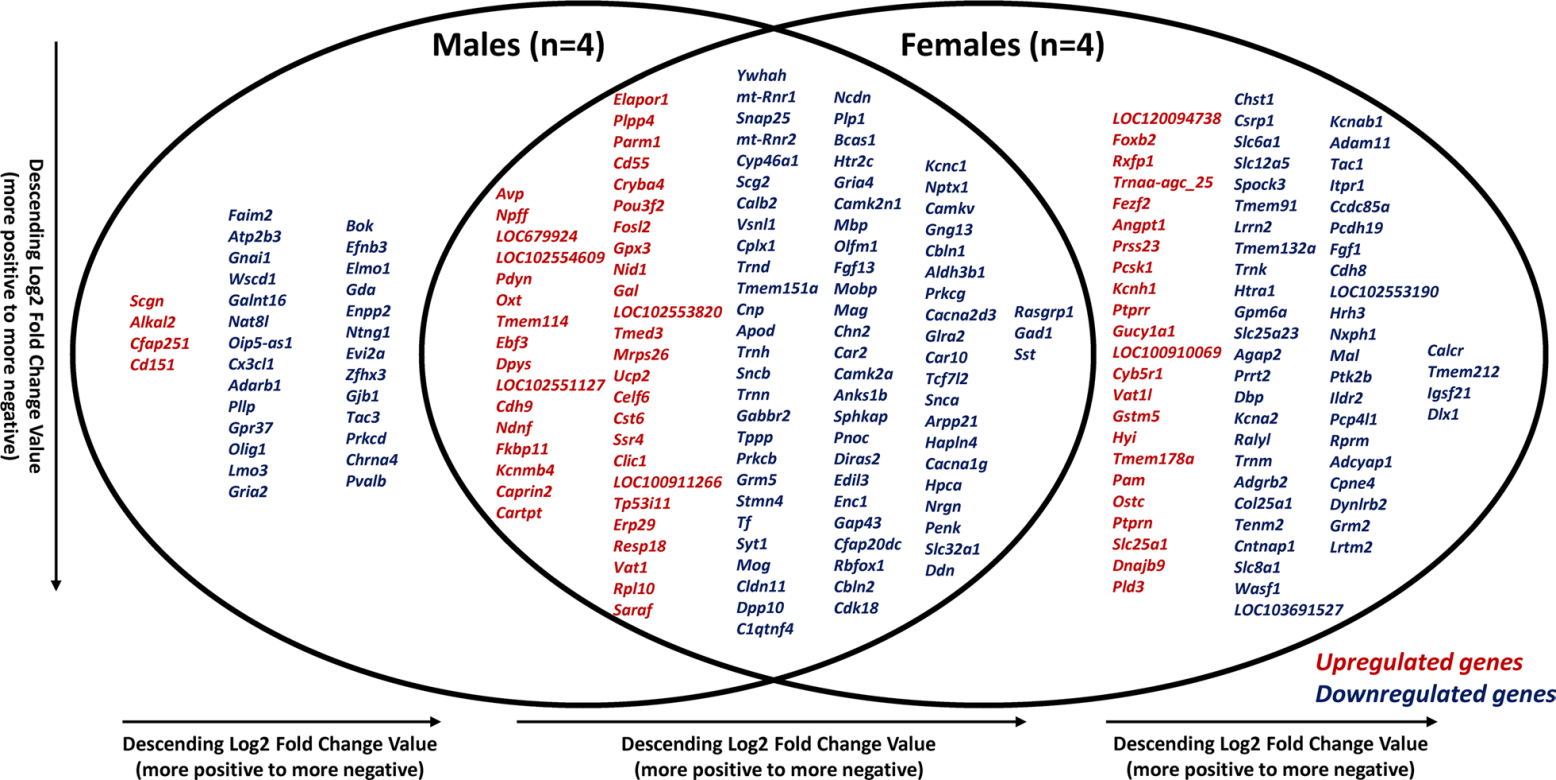
Loupe Browser DE results. Venn diagram of Loupe Browser DE results showing 220 DEGs: 116 genes common genes to both groups (e.g., *Avp, Oxt*), 31 genes unique to the male group (*n* = 4), and 73 genes unique to the female group (*n* = 4). Red indicates upregulated genes while blue indicates downregulated genes. For this analysis, upregulated and downregulated refers to genes with either increased expression (positive log2-fold change) or decreased expression (negative log2-fold change) in the SON as compared to the data from rest of the brain section. For each set, DEGs were arranged in order of descending log2-fold change value (more positive to more negative)

A total of 116 transcripts were identified as significant SON DEGs common to both male and female rats (Fig. 4). Of these 116 significant DEGs, 41 DEGs were upregulated and 75 DEGs were downregulated. In male rats, 31 significant SON DEGs were identified to be unique to this group. In this set, 4 were upregulated and 27 were downregulated. For the female rats, 73 significant SON DEGs were identified as unique to the females, in which 23 of those DEGs were upregulated and 50 DEGs were downregulated. Upregulated and downregulated in the context of Loupe Browser-identified DEGs refers to genes with increased expression (positive log2-fold change) and genes with decreased expression (negative log2-fold change), respectively, in the SON compared to the rest of the brain section. Among these, transcripts encoding the oxytocin (*Oxt*) and vasopressin (*Avp*) genes were of the most highly expressed in the SON based on median-normalized average count values from the eight samples. The means (*n* = 8) of the median-normalized average counts for *Oxt* and *Avp* were 1037.87 and 1021.71, respectively, which were magnitudes higher than the value for the next highest significant DEG common to males and females (*mt-Rnr2*, 44.23).

**Fig. 5:**
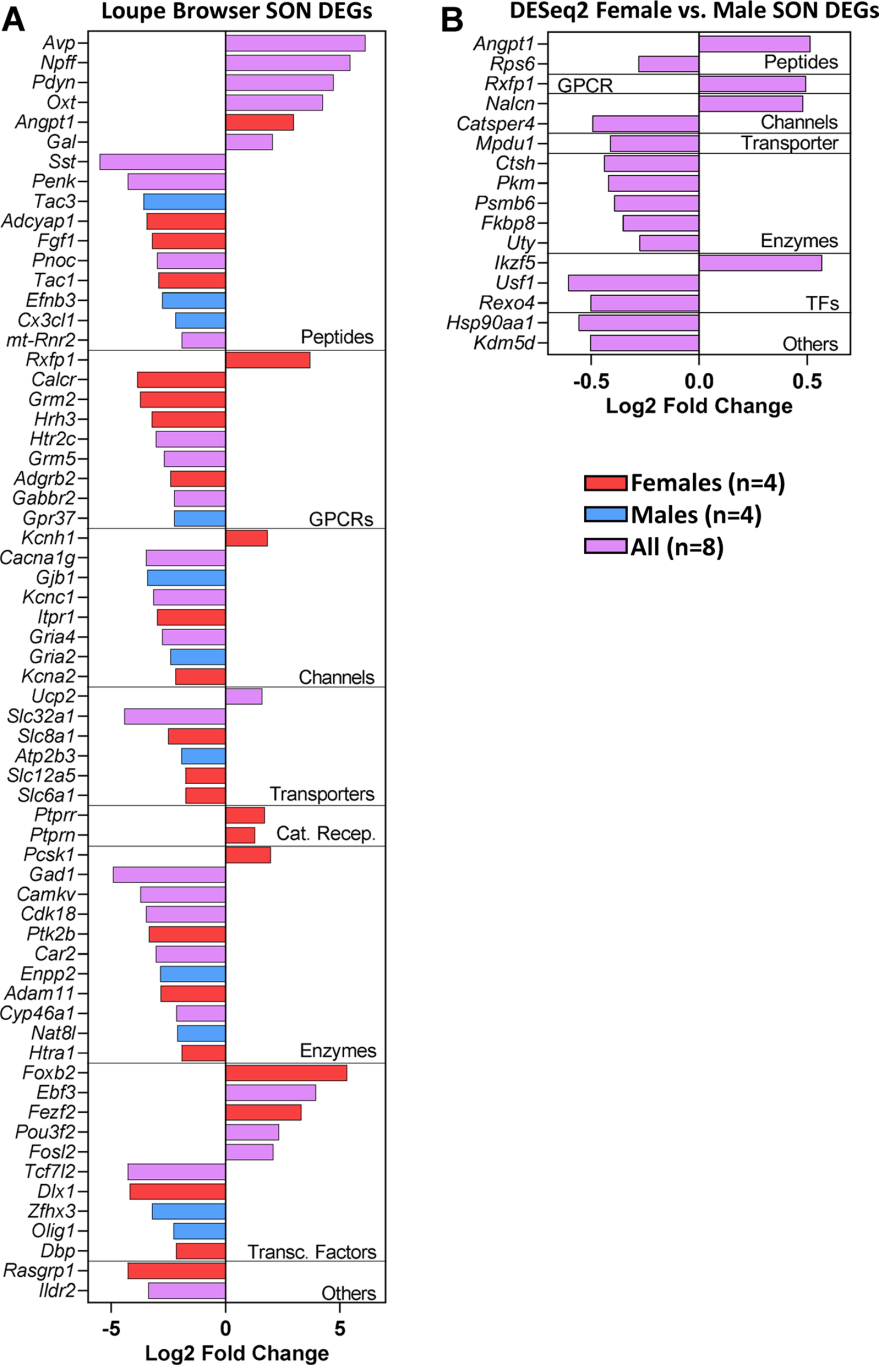
IUPHAR and transcription factors classifications of SON DEGs based on Loupe Browser DE analysis (**A**) and DESeq2 DE analysis (**B**). Red, females (*n* = 4); blue, males (*n* = 4); lilac, both males and females (*n* = 8)

These transcripts were further analyzed using IUPHAR and transcription factor classifications. The major categories identified and representative transcripts for each category are illustrated in Fig. 5A. The Peptide category included some transcripts that were upregulated such as *Avp,* which was the top expressed peptide in both males and females, followed by neuropeptide FF-amide peptide precursor *(Nffp),* prodynorphin* (Pdyn),* angiopoietin 1 *(Angpt1*, only in females*), Oxt,* and galanin (*Gal*). Most of the genes except for two in Catalytic Receptors and one each in GPCRs, Channel, Transporter, and Enzyme categories were downregulated, whereas the genes in the Transcription Factors category had a more equal number of upregulated vs. downregulated genes. The upregulated transcripts included a relaxin family peptide receptor 1 (*Rxfp1*) in GPCRs, a potassium voltage-gated channel subfamily H member 1 (*Kcnh1*) in Channels, protein tyrosine phosphatase receptors types R (*Ptprr*) and N (*Ptprn*) in Catalytic Receptors, and proprotein convertase subtilisin/kexin type 1 (*Pcsk1*) in Enzymes, which were all specific for females. In the Transporter category, uncoupling protein 2 (*Ucp2*) was upregulated in both males and females. Many of the downregulated transcripts in each category were related to neuronal function or neurotransmitter systems. These include *Calcr*, *Grm2*, and *Hrh3* in GPCRs; *Slc32a1*, *Slc12a5*, and *Slc6a1* in Transporters; *Cacna1g*, *Gria4*, and *Gria2* in Channels; and *Gad1, Camkv,* and *Cdk18* in Enzymes. DEGs encoding for transcription factors that were upregulated in both males and females include *Ebf3, Pou3f2*, and *Fosl2,* while *Tcft7l2* was the only downregulated gene encoding transcription factor common to both groups. There were 4 DEGs for transcription factors specific for females of which two were upregulated (*Foxb2, Fezf2*) while two were downregulated *(Dlx1, Dbp*) and two downregulated DEGs specific to males (*Zfhx3, Olig1*).

#### Significant SON DEGs determined using DESeq2

**Fig. 6:**
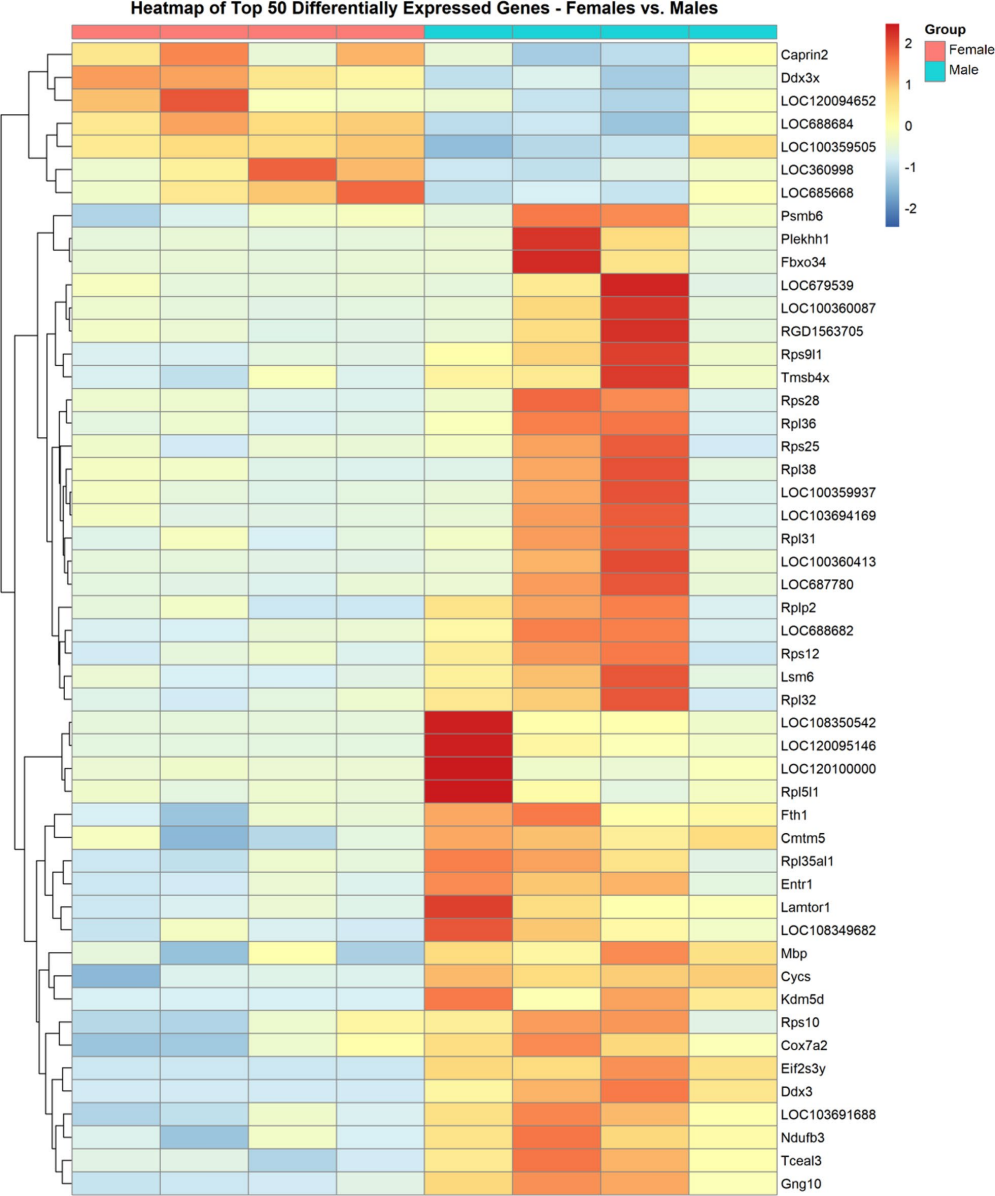
Top 50 SON DEGs from DESeq2. Clustered heatmap displaying top 50 SON DEGs from DESeq2 comparing female group (*n* = 4, coral) vs. male group (*n* = 4, teal)

DESeq2 identified 183 DEGs (nominal *p*-value < 0.05). Of these 183 DEGs, 42 genes were upregulated and 141 genes were downregulated in SON of female rats as compared to male rats. Upregulated and downregulated in the context of DESeq2 refers to genes differentially expressed (increased or decreased, respectively) in females compared to the males. IUPHAR and transcription factors classifications revealed 2 genes in the Peptide category (*Angpt1 and Rps6*), 1 upregulated gene in the GPCR category (*Rxfp1*), 2 channel-related genes (*Nalcn and Catsper4*), 1 downregulated transporter (*Mpdu1*), 5 downregulated genes in the Enzymes category, 3 genes in the Transcription Factors category (*Ikzf5, Usf1, Rexo4*), and 2 downregulated genes in the Other Targets (Fig. 5B). A clustered heatmap analysis was performed and results for the top 50 SON DEGs in females vs. males are shown in Fig. 6. The *Caprin*2 and *Ddx3x* were among the top upregulated SON DEGs, while genes for ribosomal proteins (e.g., *Rps25, Rps28, Rpl36, Rpl38*) were among the top downregulated SON DEGs in females when compared to males.

#### Comparison of significant SON DEGs determined using Loupe Browser and DESeq2

The IUPHAR and transcription factor classifications in Fig. 5 provided a snapshot comparison between the results of the two DE analysis approaches. Further integrating the results from the two DE methods, comparisons were made between the Loupe Browser DEGs unique to female and male groups with DEGs identified by DESeq2 Female vs. Male (Additional file 2: Fig. S18). Four DEGs (i.e., *Angpt1*, *Cyb5r1*, *Rxfp1*, and *Vat1l*) were found to be common between the 73 Loupe Browser DEGs unique to the females and the 183 DESeq2 Female vs. Male DEGs. All four genes were found upregulated in the respective DE analyses (Additional file 2: Fig. S18). No genes were found to be common between the 31 DEGs identified via Loupe Browser unique to the male group and the 183 DESeq2 Female vs. Male DEGs.

### Gene Ontology and pathway analysis of SON DEGs

#### GO terms/pathways for significant SON DEGs identified using Loupe Browser

**Fig. 7:**
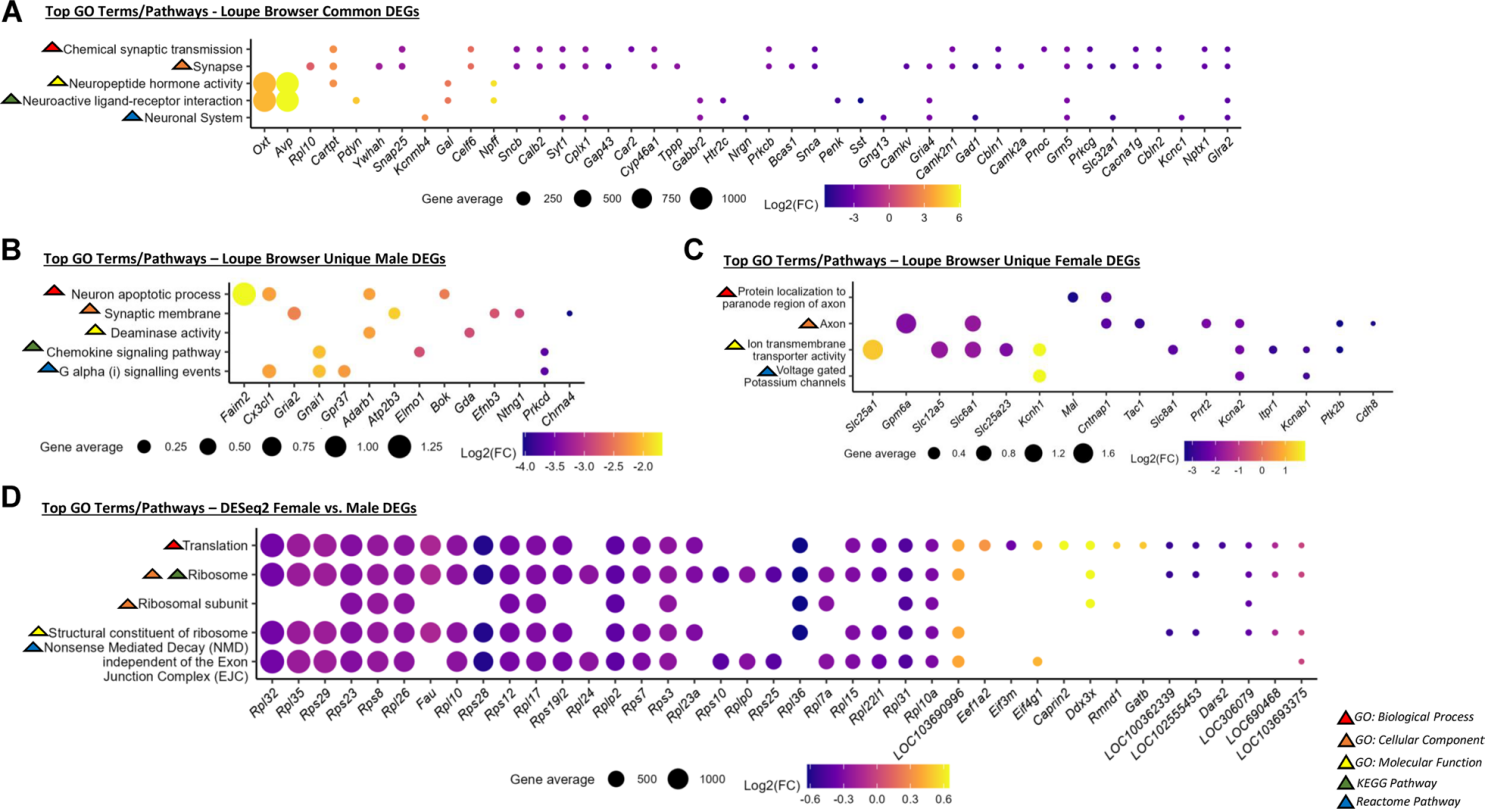
Top GO enrichment analysis terms and pathways. Top GO terms and pathways per category are shown for **A** Loupe Browser-identified SON DEGs common to both sexes (*n* = 8), **B** Loupe Browser-identified SON DEGs unique to males (*n* = 4), **C** Loupe Browser-identified SON DEGs unique to females (*n* = 4), and **D** DESeq2-identified SON DEGs in females (*n* = 4) compared to males (*n* = 4). Dot size corresponds to gene average size and dot color represents log2-fold change. Categories for GO terms and pathways represented by colored triangles: red = GO: Biological Process, orange = GO: Cellular Component, yellow = GO: Molecular Function, green = KEGG pathway, and blue = Reactome pathway

Using the list of significant DEGs from the SON common to both males and females, GO enrichment and pathway analyses revealed terms/pathways related to neuropeptide hormone and synaptic transmission. The top hits for the five GO/pathway categories included ‘Chemical synaptic transmission’, ‘Synapse’, ‘Neuropeptide hormone activity’, ‘Neuroactive ligand-receptor’, and ‘Neuronal System’ (Fig. 7A). *Oxt* and *Avp* had the largest gene averages by magnitudes greater than the other genes contributing to the terms/pathways. The top terms/pathways for SON DEGs unique to the males were more related to cell signaling (‘Neuron apoptotic process’, ‘Synaptic membrane’, ‘Deaminase activity’, ‘Chemokine signaling pathway, and ‘G alpha (i) signaling events’; Fig. 7B). For the females, the top terms/pathways were more associated with axons (‘Protein localization to the paranode region of axon’ and ‘Axon’) and membrane transport (‘Ion transmembrane transporter activity’ and ‘Voltage gated Potassium channels’; Fig. 7C). Additional plots, gene input lists, and a complete list of GO enrichment/pathway analysis results for Loupe Browser-identified SON DEGs are available in the Additional file 2: Figs. S19-21, 23-25 and Additional files 6 and 8.

#### GO terms/pathways for significant SON DEGs in females vs. males via DESeq2

The GO enrichment and pathway analyses using the list of significant SON DEGs in females versus males using DESeq2 identified top hits per GO term/pathway categories related to ribosomes and ribosomal pathways (‘Translation’, ‘Ribosome’, ‘Ribosomal subunit’, ‘Structural constituent of ribosome’, and ‘Nonsense Mediated Decay (NMD) independent of the Exon Junction Complex (EJC)’; Fig. 7D). ‘Ribosome’ was the top hit in both GO: Cellular Component and KEGG pathway categories. Additional plots, gene input lists, and a complete list of GO enrichment/pathways analysis results for DESeq2-identified SON DEGs are also available in Additional fil 2: Figs. S22, 26 and Additional files 7 and 9.  It should be noted that the range of the log2-fold changes observed for DESeq2 SON DEGs was much smaller than the ranges for the log2-fold changes observed for the data sets from Loupe Browser.

### IPA results

**Fig. 8:**
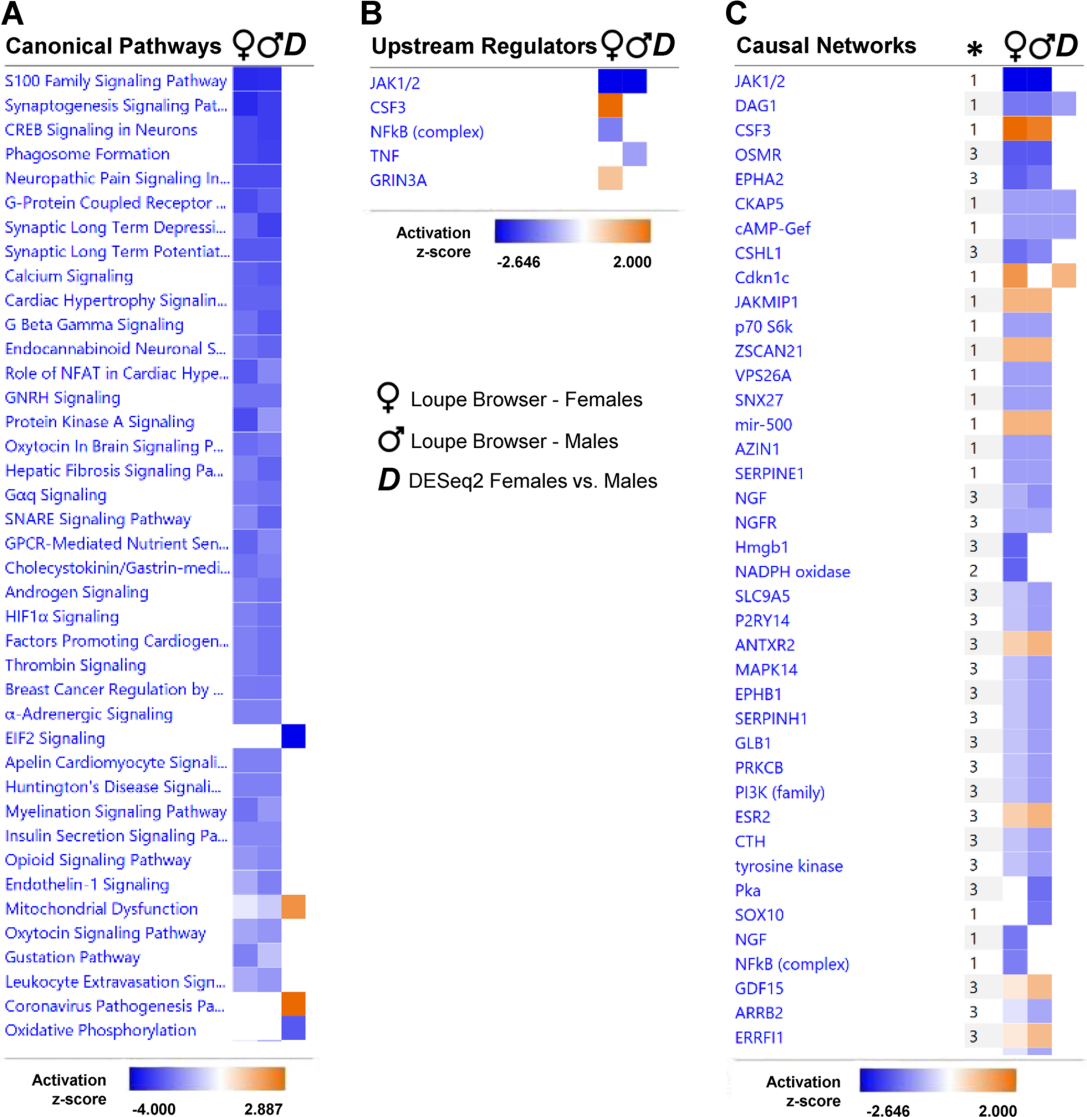
IPA Comparisons. IPA comparisons were made between first 40 canonical pathways (**A**), upstream regulators (**B**), and first 40 causal networks (**C**) identified for significant DEGs from Loupe Browser female group (*n* = 4),Loupe Browser male group (*n* = 4), and DESeq2 (female vs. male group). * indicates causal network depth according to the degree of separation between the upstream regulator and downstream target molecules in the data set

In addition to GO enrichment and pathway analysis of the SON DEGs, we also performed QIAGEN Ingenuity Pathway Analysis to further explore canonical pathways, predicted upstream regulators, and causal networks. A summary of the side-by-side comparisons between the IPA results for the significant SON DEGs lists from Loupe Browser and DESeq2 DE methods identified differences in results based on the two different approaches. For example, the DESeq2 method identified four pathways in the top 40 canonical pathways, two predicted to be activated (‘Mitochondrial Dysfunction’ and ‘Coronavirus Pathogenesis’) and two inhibited (‘EIF2 Signaling’ and ‘Oxidative Phosphorylation’; Fig. 8A). In contrast, more canonical pathways were identified using DEGs from Loupe Browser (Fig. 8A). All of the Loupe Brower determined top 40 canonical pathways had negative z-scores indicating probable downregulation. Many of these pathways are related to neural function such as ‘Synaptogenesis Signaling’, ‘Synaptic Long-Term Depression’, ‘Synaptic Long-Term Potentiation’, ‘Oxytocin Brain Signaling’, and ‘SNARE Signaling’. While no predicted upstream regulators were associated with the DESeq2 results, five upstream regulators were predicted based on the Loupe Browser results (Fig. 8B). Specifically, TNF was identified as being "inhibited” in males. In contrast, the NFKB complex was identified as being “inhibited” specifically in females. In addition, GRIN3A and CSF3 were identified as being “activated” and specific to females. Although the results of canonical pathways and upstream regulators showed little overlap among the results for DESeq2 and Loupe Browser, three causal networks (DAG1, CKAP5, and cAMP-Gef) were indicated to be “inhibited” in all three data sets (Fig. 8C). An expanded list of IPA Comparison Analysis results is available in Additional file 2: Fig. S27.

## Discussion

Using new spatial transcriptomics technology, we were able to obtain the whole transcriptomic data from coronal brain sections containing the SON from adult male and female Sprague–Dawley rats. This was accomplished by using the specially-designed Visium slides from 10x Genomics, with each slide containing four capture areas defined by unique fiducial frames, each capture area containing approximately 5000 barcoded spots, and each barcoded spot lined with millions of spatially-barcoded oligonucleotide probes with a unique molecular identifier. Essentially, this creates a system where each unique oligonucleotide probe can be traced back to a barcoded spot related to a specific address on the slide. Aligning the digital images of the H&E-stained hypothalamic brain sections to digital maps of the barcoded spots enabled us to identify barcoded spots located within the SON region. The transcripts associated with barcoded spots located within the anatomically defined SONs were used for subsequent analyses.

The validity of our results is supported by both internal findings as well as in comparison to current literature on SON transcriptomics that utilized more traditional microarray/bulk RNAseq data. Some of these internal findings from our current Visium study include: (1) gene cluster analysis successfully differentiated barcoded spots overlaying regions of nuclei from those overlaying areas of myelinated fiber tractsand (2) *Oxt* and *Avp* were the top two highly expressed genes by magnitudes in the SON. This indicates that the hypothalamic regions identified from the different brain sections using Loupe Browser successfully included the magnocellular neurons in the SON. Additionally, spatial gene expression analysis targeting *Avp* and *Oxt* using Loupe Browser revealed the barcoded spots in the SON with the highest expression of the two genes had an anatomical distribution consistent with the literature (i.e., the predominantly *Oxt*-expressing neurons tended to be located in the dorsal SON while the predominantly *Avp*-expressing neurons were located more in the ventral SON) [4, 5]. This distribution is reported in one paper that used female rats [4] while the other paper failed to report the sex of the rats [5]. With the dorsal/ventral distribution present in both the female and male rats in our study, this could potentially be the first demonstration of this anatomic distribution of *Avp* and *Oxt* neurons in the SON of both sexes using spatial transcriptomics. Our current data were also significantly correlated with the results from SON transcriptomics data from bulk RNAseq [19] and a separate proteomics study [34].

Two different differential expression analysis methods were used to analyze the spatial transcriptomics data from the SON. In the first method, Loupe Browser was used to identify genes enriched in the SON of each sample as compared to the rest of their respective sections. While this approach did not allow us to directly compare the data from the four males to the data from the four females, we were able to identify many differentially expressed genes that were specific to males and females and common to both sexes. As mentioned above, two of the transcripts that were significantly expressed in both males and females were *Avp* and *Oxt.* Other peptides that were differentially expressed in both males and females were *Pdyn, Nffp,* and *Gal.* These peptides have been shown to be expressed in MNCs and modify the electrophysiological activity of MNCs [42–45]. Other transcripts that have been linked to MNCs function and were differentially expressed in both sexes include *Caprin2*, *Gabbr2*, *Slc32a1*, and *Cartpt.* The list of significant differentially expressed transcripts specific to female rats included several transporters (*Slc12a5*, *Slc6a1*, and *Slc25a23*), peptides (*Angpt1, Tac1*, and *Adycap1*), and two tyrosine phosphatase receptors (*Ptprn* and *Ptprr*). DEGs that were specific to males also included genes related to neural function such as *Chrna4*, *Gria2*, *Gnai1*, and *Tac3*. A complete list of the significant SON DEGs that were identified through Loupe Browser can be seen in Fig. 4. While there were some differences among the SON DEGs between the males and females, several of the transcripts were described by the same IUPHAR classification terms and most of these transcripts were downregulated as referenced against the rest of their sample (Fig. 5A).

Although the DESeq2 analysis was significantly correlated with the results of the Loupe Browser-based analysis, there was little apparent overlap in the list of differentially expressed genes in female rats compared to male rats. Several of the transcripts among the top 50 DEGs identified using the DESeq2 method were ribosomal proteins. These differences were clearer in the results of the GO terms and IPA analyses. The GO term/pathway analysis of DEGs from Loupe Browser common to males and females, male-specific, and female-specific generally resulted in terms related to neuronal function and cell signaling. For example, the top GO terms identified using the transcripts through the Loupe Brower approach were ‘Neuropeptide Hormone Activity’ (Fig. 7A), ‘Neuron Apoptotic Processes’ (Fig. 7B), and ‘Ion transmembrane Transporter Activity’ (Fig. 7C), respectively. In contrast, the top GO terms associated with the DESeq2 analysis were ‘Translation’, ‘Ribosome’, and ‘Ribosomal Subunit’ (Fig. 7D). The IPA analysis results identified a number of canonical pathways and causal networks in the Loupe Browser data while fewer canonical pathways and causal networks were associated with the DESeq2 results (Fig. 8).

As mentioned previously, Loupe Browser is currently limited to analyzing individual capture areas for the Visium application. Therefore, it was not possible to make comparisons between samples in different capture areas on the same slide or across different slides with the version of Loupe Browser that we used. This study used a workflow that attempted to address these limitations. However, it should be noted that the expression values per group were not normalized across samples in the traditional sense. Instead, the data were averaged. This approach does not affect the GO and pathway analyses because the data inputs were based on the DEGs lists and not expression values. This may not be true for the Loupe Browser method-based IPA analysis.

Another important difference is that Loupe Browser’s globally distinguishing Significant Feature Comparison Analysis compares region(s) of interest to the rest of the data set from that specific capture area. Thus, Loupe Browser data for each SON are dependent on data from the rest of the barcodes excluding the SON-associated barcoded spots in that capture area. Although precautions were taken to get the sections as close to the same size and from the same coronal plane of the hypothalamus, some variation was unavoidable. The anatomical variation could potentially confound the Loupe Browser DE results. One of the main reasons that we conducted a second DE analysis method with DESeq2 was to help determine the impact of this Loupe Browser limitation on the results. We also wanted to use a more traditional DE analysis workflow since it has a built-in normalization step and would allow us to more directly make comparisons of baseline gene expression for SONs from female vs. male adult rats.

These different workflows yielded different results both in terms of sex differences in the individual transcripts that are differentially expressed between males and females and the global cellular process that these transcripts represent. Only four DEGs were found to be common between the two DE analysis approaches. All four genes (i.e., *Angpt1*, *Cyb5r1*, *Rxfp1*, and *Vat1l*) were upregulated both in the list for Loupe Browser DEGs unique to the female group and for DESeq2 Female vs. Male DEGs. This minimal overlap in the DEGs was not surprising, as again, the two DE analysis approaches are making comparisons using different aspects of the data sets. With Loupe Browser, the comparison is made between the region of interest (i.e., the SON) to the rest of the section for each respective sample to determine DEGs, while the DESeq2 approach more directly compares the SON gene expression between the females and males to identify DEGs. These two different approaches were used together to provide a more holistic picture of sex differences in SON gene expression in adult rats and to better understand the limitations of each approach in the context of spatial transcriptomics.

Our results identified several transcripts that could be involved in sex differences in the physiological regulation of AVP and OXT synthesis and release. For example, *Caprin2* was among the differentially expressed transcripts common to both males and females according to the Loupe Browser analysis while the DESeq2 results indicated that this transcript was upregulated in females as compared to males. *Caprin2* encodes an RNA-binding protein and, in male rats, *Caprin2* binds to AVP mRNA and regulates the length of its poly(A) tail [46]. When rats are stimulated by systemic hyperosmolality, such as chronic salt loading and water deprivation [19, 47], the expression of *Caprin2* is highly increased in the SON. In rats submitted to hyperosmolality induced by water deprivation, *Caprin2* is strongly hyperphosphorylated in the SON [34] and its expression increases in both AVP and OXT MNCs [48]. Knockdown of *Caprin2* in the SON of dehydrated male rats attenuated the normal increase in AVP mRNA but paradoxically was also associated with increased plasma AVP [46]. Following sodium depletion, *Caprin2* expression was decreased in the SON [49]. It should be noted that these studies used only male rats. Based on our results, it is possible that *Caprin2* could contribute to sex differences in the expression and secretion of AVP and OXT mRNA in female rats.

The transcript for *Slc12a5* was identified as downregulated in the SON of females as compared to males. *Slc12a5* is the gene for the potassium-chloride cotransporter 2 (KCC2) which extrudes chloride from the cell to maintain the chloride concentration gradient across the cell membrane in neurons [50]. While the expression of *Slc12a5* in AVP neurons has been controversial [51, 52], the current results indicate it is expressed in SON. This is consistent with in vitro studies which showed that changes in KCC2 function contribute to a shift in the chloride equilibrium protentional in AVP SON neurons from dehydrated male rats [51, 53]. However, the expression of *Slc12a5* appears to be lower in females. This could suggest that changes in the phosphorylation status of KCC2 that contribute to alterations of the reversal potential for chloride in MNCs may have a greater impact in males as compared to females.

Tachykinins are known to play a major role in reproductive function [54]. Despite being previously identified as expressed in the SON [55, 56], the physiological role of the tachykinin peptides associated with the SON remains to be determined. *Tac1* encodes a pro-peptide that, when processed, generates substance P, neuropeptide gamma, neurokinin A, and neuropeptide K—four products of the tachykinin peptide family. On the other hand, *Tac3* encodes neurokinin B, another tachykinin family peptide [57]. Our spatial transcriptomics results identified *Tac1* as differentially expressed in the SON of female but not male rats. In contrast, *Tac3* is differentially expressed in the SON of male but not female rats. This suggests that tachykinin peptides could contribute to sex differences in the regulation of AVP and OXT secretion.

In higher order analyses of our results, GO term/pathway analysis of DEGs common to both males and females as determined by Loupe Browser identified terms related to neural function such as neuropeptide activity and chemical synaptic transmission. Similarly, the GO terms specific for males and females were also focused on neurophysiological functions. In contrast, the GO terms derived using the DESeq2 DEGs were heavily focused on translation, ribosomes, and ribosomal subunits. These latter findings are more consistent with previous GO term analyses [23]. The GO: Molecular Function terms and KEGG terms associated with the Loupe Browser DEGs were also identified by bulk RNA sequencing the SON of dehydrated male rats [19]. The results from the Ingenuity Pathway Analysis were similar in that the Loupe Browser analysis identified more pathways than the DESeq2 analysis and that the majority of the Loupe Browser pathways were associated with neural function. This suggests some consensus in the results obtained through different sequencing approaches and most of the terms are related to known functions of MNCs. The sex differences related to translation and ribosomal function could be considered unexpected. Given the changes associated with the function of OXT neurons during lactation and parturition, it could be argued that female SON would require a greater capacity for protein synthesis than the SON of male rats. Alternatively, these results were obtained from virgin male and female rats. Different results might be obtained from males and females with sexual experience or pregnant or lactating female rats.

Both AVP and OXT neurons demonstrate structural and functional adaptions to physiological challenges such as dehydration [58–60], changes in physiological state such as pregnancy [59, 61], and pathophysiological states such as hypertension or hyponatremia [53, 62, 63]. These results indicate that SONs from females and males express transcripts associated with canonical pathways related to plasticity (e.g., synaptogenesis, long-term depression (LTD), long-term potentiation (LTP)). However, the results of the IPA analysis indicate that these plasticity-related pathways are inhibited relative to other brain regions in the same sections. The SON is capable of both LTP and LTD [64] and structural plasticity that includes astrocytes [9, 65]. It is possible that the observed inactivation state represents the basal status of the SON and that upregulation of the genes associated with these terms would require a change in the physiological state of the animal.

## Limitations

Although we were able to generate gene expression profiles from whole transcriptomic data from anatomically defined SONs from male and female rats, the results were limited to cells located on barcoded spots underlying the brain sections containing the SON of male and female rats. There are some limitations to consider with this approach. With each barcoded spot being 55 µm in diameter and a center-to-center distance of 100 µm between adjacent spots, the current resolution of the Visium Spatial Gene Expression technology is approximately 1–10 cells, depending on the tissue sample. Magnocellular neurons in the SON are larger than most cell types, averaging 40 µm in diameter [1, 2]. In theory, the size of MNCs in SON would allow the results to better approximate single cell resolution. The results were also dependent on how the MNCs aligned with the barcoded spots. The placement of the tissue section also matters due to the spacing of the barcoded spots. The specific alignment of MNCs with barcoded spots was difficult to control during sample preparation and could not be determined until data analysis. Nevertheless, the number of barcoded spots located within each SON was fairly even across the samples. While our results are consistent with previous studies of the SON transcriptome and proteome, their correspondence to single-cell or single-nucleus RNAseq data cannot be determined until such data are published.

Another limitation is that the female rats included in the current study were only tested once at the end of the study to determine their stage in the estrous cycle. Three of the four females were in proestrus and one was in metestrus/diestrus, but it is possible that they were not normally cycling. This left us unable to test the effects of the estrous cycle on SON gene expression. Changes in ovarian hormones do affect gene expression in several brain regions including the hypothalamus [66]. Further studies will be needed to determine the effects of the estrous cycle on SON gene expression using spatial transcriptomics.

## Perspectives and significance

The current spatial transcriptomics study revealed basal sex differences in SON gene expression in adult rats related to cell signaling and ribosomal pathways. Future spatial transcriptomics will extend this analysis to other regions contained in these sections. It would also be of interest to determine if these results provide new information related to the function of AVP and OXT neurons in the SON. Previous studies in our lab have found sex differences in SON secretion of vasopressin and oxytocin in a model of dilutional hyponatremia [24]. We would like to explore this further with spatial transcriptomics to gain insight into genes that contribute to the pathophysiology and functional sex differences in the SON.

### Supplementary Information

**Additional file 1.** List of additional files and figures.

**Additional file 2.** Supplemental figures 1–27.

**Additional file 3.** **Spreadsheet 1.** SON DEGs for Loupe Browser Females (Less Conservative).

**Additional file 4.** **Spreadsheet 2.** SON DEGs for Loupe Browser Males (Less Conservative).

**Additional file 5.** **Spreadsheet 3.** SON DEGs for DESeq2 Females vs. Males.

**Additional file 6.** **Spreadsheet 4.** Inputs for GO/Pathway Analyses Obtained from Loupe Browser Less Conservative Approach.

**Additional file 7.** **Spreadsheet 5.** Inputs for GO/Pathway Analyses Obtained from DESeq2 Females vs. Males.

**Additional file 8.** **Spreadsheet 6.** Compiled GO Terms/Pathway Results for Loupe Browser Less Conservative Approach.

**Additional file 9.** **Spreadsheet 7.** Compiled GO Terms/Pathway Results for DESeq2 Females vs. Males.

**Additional file 10.** **Spreadsheet 8.** SON DEGs for Loupe Browser Females (Conservative).

**Additional file 11.** **Spreadsheet 9.** SON DEGs for Loupe Browser Males (Conservative).

**Additional file 12.** **Spreadsheet 10.** Inputs for GO/Pathway Analyses Obtained from Loupe Browser Conservative Approach.

**Additional file 13.** **Spreadsheet 11.** Compiled GO Terms/Pathway Results for Loupe Browser Conservative Approach.

## Data Availability

In addition to data reported in the manuscript and Additional files, all raw data files are available through the NCBI Gene Expression Omnibus (GEO) platform (Accession GSE242058 https://www.ncbi.nlm.nih.gov/geo/query/acc.cgi?acc=GSE242058).
