# Supplementary material for: Spatial transcriptomics reveal basal sex differences in supraoptic nucleus gene expression of adult rats related to cell signaling and ribosomal pathways
Source: Biol Sex Differ. 2023 Oct 19;14:71. doi: 10.1186/s13293-023-00554-3 (PMC10585758; doi:10.1186/s13293-023-00554-3)
Supplement: Supplementary file 1 — Additional file 1. List of additional files and figures. [file 13293_2023_554_MOESM1_ESM.pdf]

The data in this paper were generated from 4 untreated male and 4 untreated female adult (3-4 months) Sprague Dawley rats. Two unique methods of differential expression (DE) analysis were used: Loupe Browser (10x Genomics proprietary software, freely available) and DESeq2 (part of Bioconductor package in R, also freely available on the internet). Loupe Browser Significant Feature Comparison has the option of filtering the DE results to either keep (“less conservative” approach) or exclude (“conservative” approach) genes with low average counts. “LCI” (low counts included) in the files below refer to results obtained via the Loupe Browser (LB) “less conservative” approach. All the files list either 1) differentially expressed genes (DEGs) and associated gene expression values identified for the supraoptic nucleus (SON) region, 2) inputs (DEGs gene list and/or log2 fold change values) for the downstream gene ontology (GO)/pathway analyses, or 3) compiled GO/pathway analyses results for the different DE methods/approaches.

List of Additional Files

Additional File 2: Supplemental Figures 1-27.

- Supplemental Figure 1. Gene Cluster Analysis of Rat Forebrain Section Containing Supraoptic Nucleus (SON). Representative image
- Supplemental Figures 2-5. Defining SON Region for Females 1-4
- Supplemental Figures 6-9. Defining SON Region for Males 1-4
- Supplemental Figures 10-13. Spatial Gene Expression Analysis for Avp and Oxt for Females 1-4
- Supplemental Figures 14-17. Spatial Gene Expression Analysis for Avp and Oxt for Males 1-4
- Supplemental Figure 18. Comparison of Loupe Browser DEGs Unique to Female and Male Groups to DESeq2 Female vs. Male DEGs.
- Supplemental Figure 19. Additional dot plots for Loupe Browser Common SON DEGs (Top 5 GO terms/pathways per category based on significant enrichment FDR)
- Supplemental Figure 20. Additional dot plots for Loupe Browser Female SON DEGs (Top 5 GO terms/pathways per category based on significant enrichment FDR)
- Supplemental Figure 21. Additional dot plots for Loupe Browser Male SON DEGs (Top 5 GO terms/pathways per category based on significant enrichment FDR)
- Supplemental Figure 22. Additional dot plots for DESeq2 Females vs. Males SON DEGs (Top 5 GO terms/pathways per category based on significant enrichment FDR)
- Supplemental Figure 23. Additional dot plots for Loupe Browser Common SON DEGs (Top 10 GO terms/pathways per category based on fold enrichment)
- Supplemental Figure 24. Additional dot plots for Loupe Browser Female-Unique SON DEGs (Top 10 GO terms/pathways per category based on fold enrichment)
- Supplemental Figure 25. Additional dot plots for Loupe Browser Male-Unique SON DEGs (Top 10 GO terms/pathways per category based on fold enrichment)
- Supplemental Figure 26. Additional dot plots for DESeq2 Females vs. Males SON DEGs (Top 10 GO terms/pathways per category based on fold enrichment)
- Supplemental Figure 27. Expanded IPA Comparison Analysis results

Additional File 3: Spreadsheet 1. SON DEGs for Loupe Browser Females (Less Conservative)

Additional File 4: Spreadsheet 2. SON DEGs for Loupe Browser Males (Less Conservative)

Additional File 5: Spreadsheet 3. SON DEGs for DESeq2 Females vs. Males

Additional File 6: Spreadsheet 4. Inputs for GO/Pathway Analyses Obtained from Loupe Browser Less Conservative Approach

Additional File 7: Spreadsheet 5. Inputs for GO/Pathway Analyses Obtained from DESeq2 Females vs. Males

Additional File 8: Spreadsheet 6. Compiled GO Terms/Pathway Results for Loupe Browser Less Conservative Approach

Additional File 9: Spreadsheet 7. Compiled GO Terms/Pathway Results for DESeq2 Females vs. Males

Additional File 10: Spreadsheet 8. SON DEGs for Loupe Browser Females (Conservative)

Additional File 11: Spreadsheet 9. SON DEGs for Loupe Browser Males (Conservative)

Additional File 12: Spreadsheet 10. Inputs for GO/Pathway Analyses Obtained from Loupe Browser Conservative Approach

Additional File 13: Spreadsheet 11. Compiled GO Terms/Pathway Results for Loupe Browser Conservative Approach
