# Supplementary material for: Spatial transcriptomics reveal basal sex differences in supraoptic nucleus gene expression of adult rats related to cell signaling and ribosomal pathways
Source: Biol Sex Differ. 2023 Oct 19;14:71. doi: 10.1186/s13293-023-00554-3 (PMC10585758; doi:10.1186/s13293-023-00554-3)
Supplement: Supplementary file 2 — Additional file 2. Supplemental figures 1–27. [file 13293_2023_554_MOESM2_ESM.pdf]

Gene Cluster Analysis

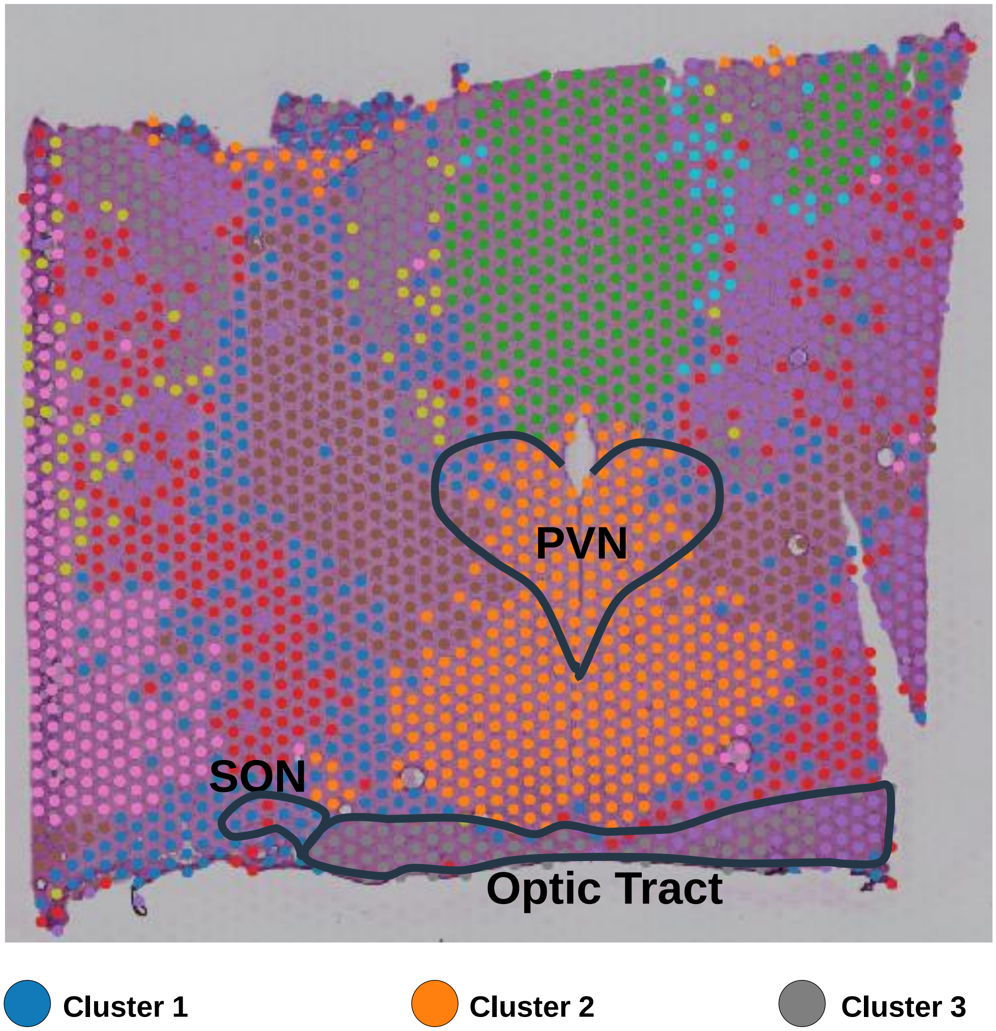

Supplemental Figure 1: Gene Cluster Analysis of Rat Forebrain Section Containing Supraoptic Nucleus (SON). Representative image of gene cluster analysis results showing successful differentiation of nuclei from myelinated fiber tracts (e.g., Cluster 3), SON clustered with MNCs from paraventricular nucleus (PVN) of the hypothalamus (Cluster 1), and parvocellular PVN was part of a different cluster (Cluster 2).

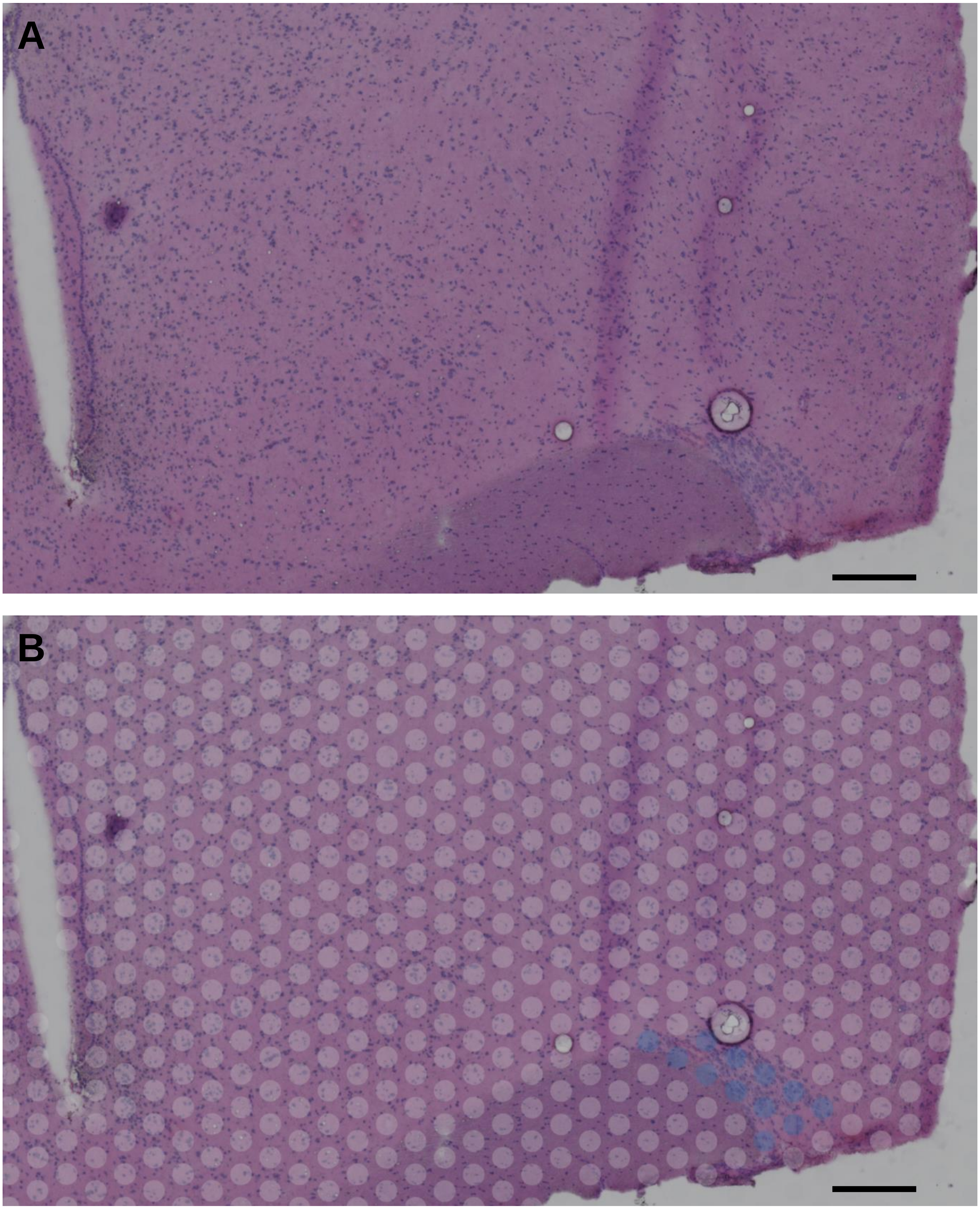

Supplemental Figure 2: Defining SON Region – Female 1. (A) Magnified hematoxylin and eosin (H&E) image of coronal brain section from Female 1. (B) Barcoded spots overlaying SON region of Female 1 selected (blue). Scale bar = 250 µm.

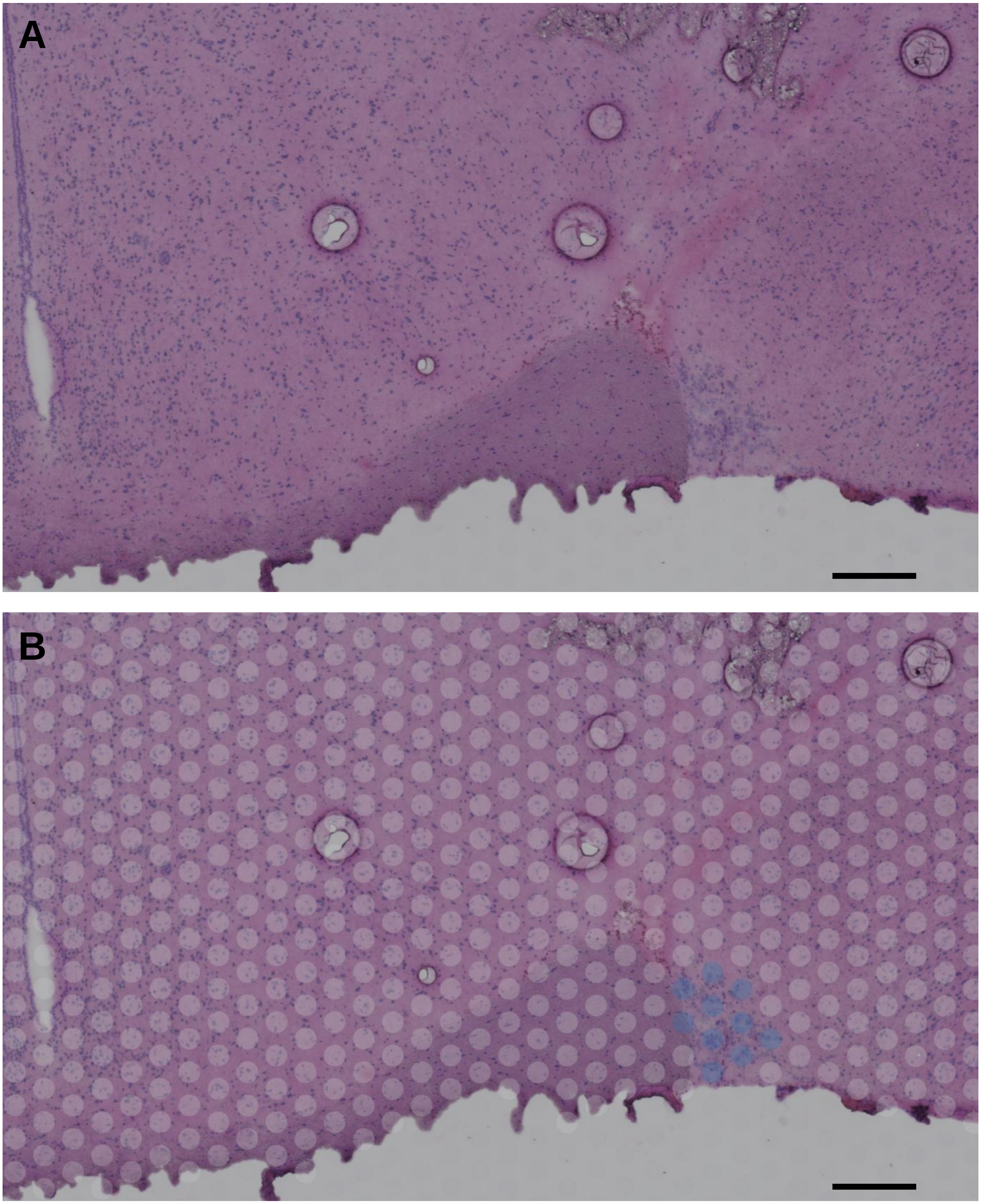

Supplemental Figure 3: Defining SON Region – Female 2. (A) Magnified H&E image of coronal brain section from Female 2. (B) Barcoded spots overlaying SON region of Female 2 selected (blue). Scale bar = 250 µm.

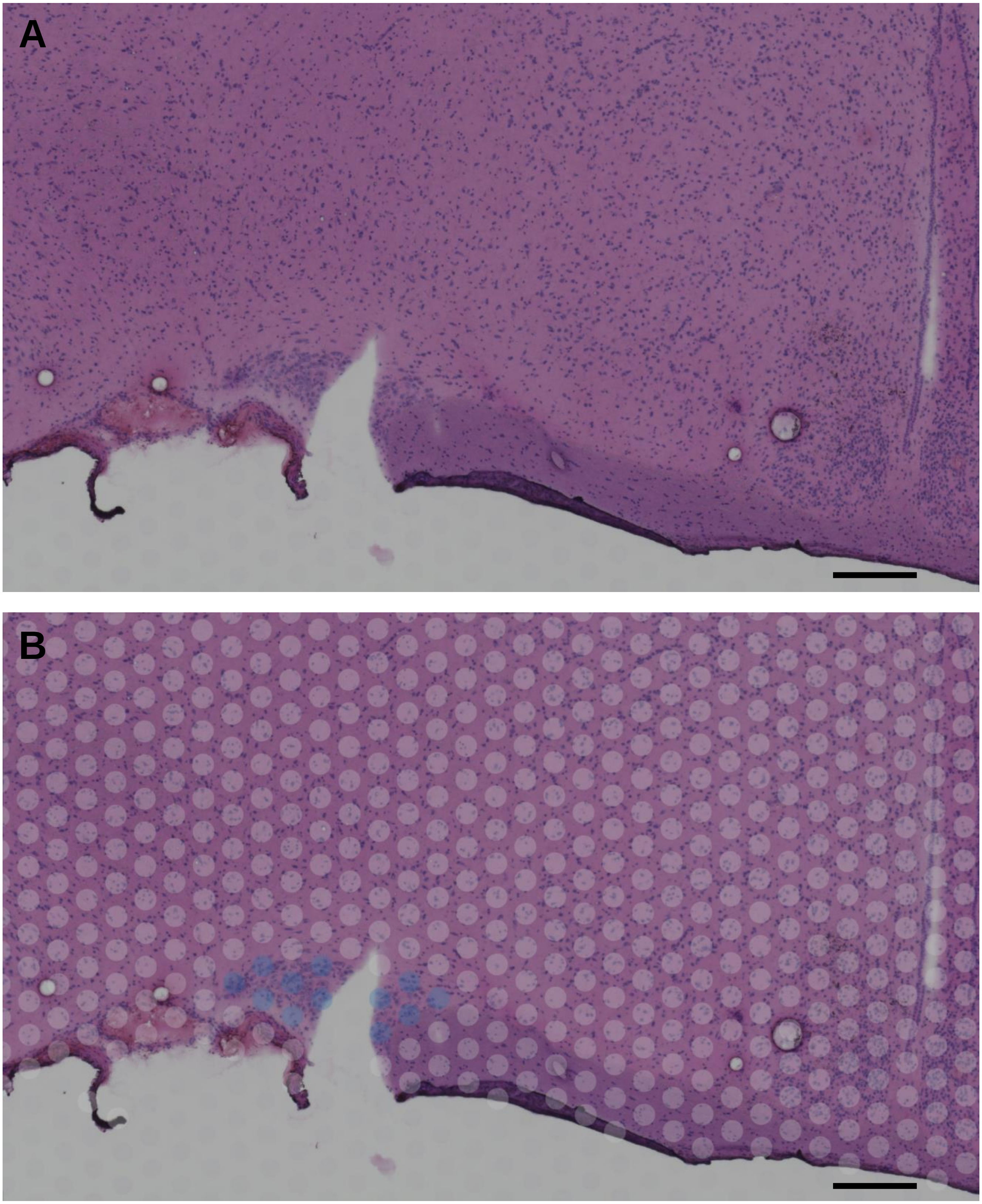

Supplemental Figure 4: Defining SON Region – Female 3. (A) Magnified H&E image of coronal brain section from Female 3. (B) Barcoded spots overlaying SON region of Female 3 selected (blue). Scale bar = 250 µm.

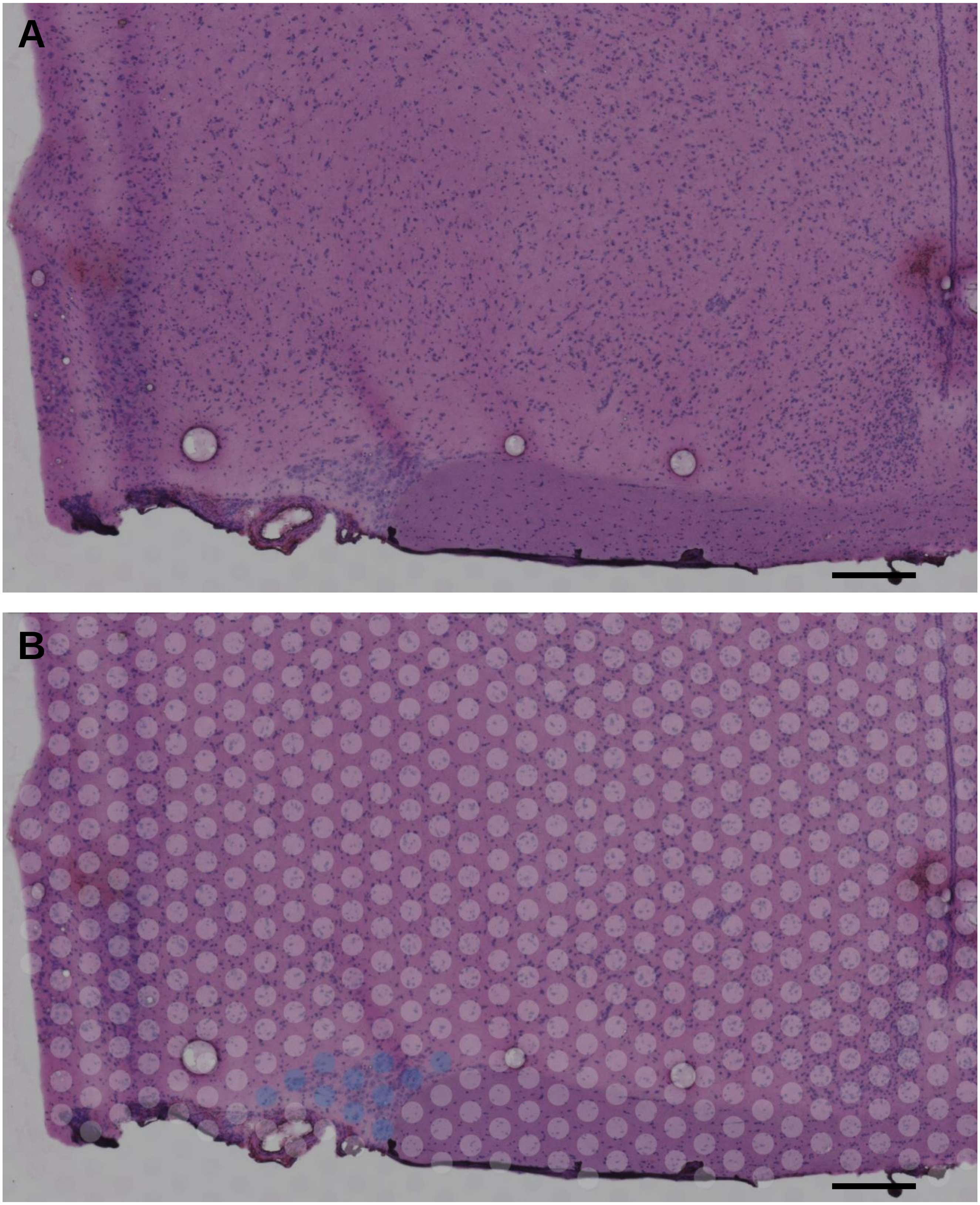

Supplemental Figure 5: Defining SON Region – Female 4. (A) Magnified H&E image of coronal brain section from Female 4. (B) Barcoded spots overlaying SON region of Female 4 selected (blue). Scale bar = 250 µm.

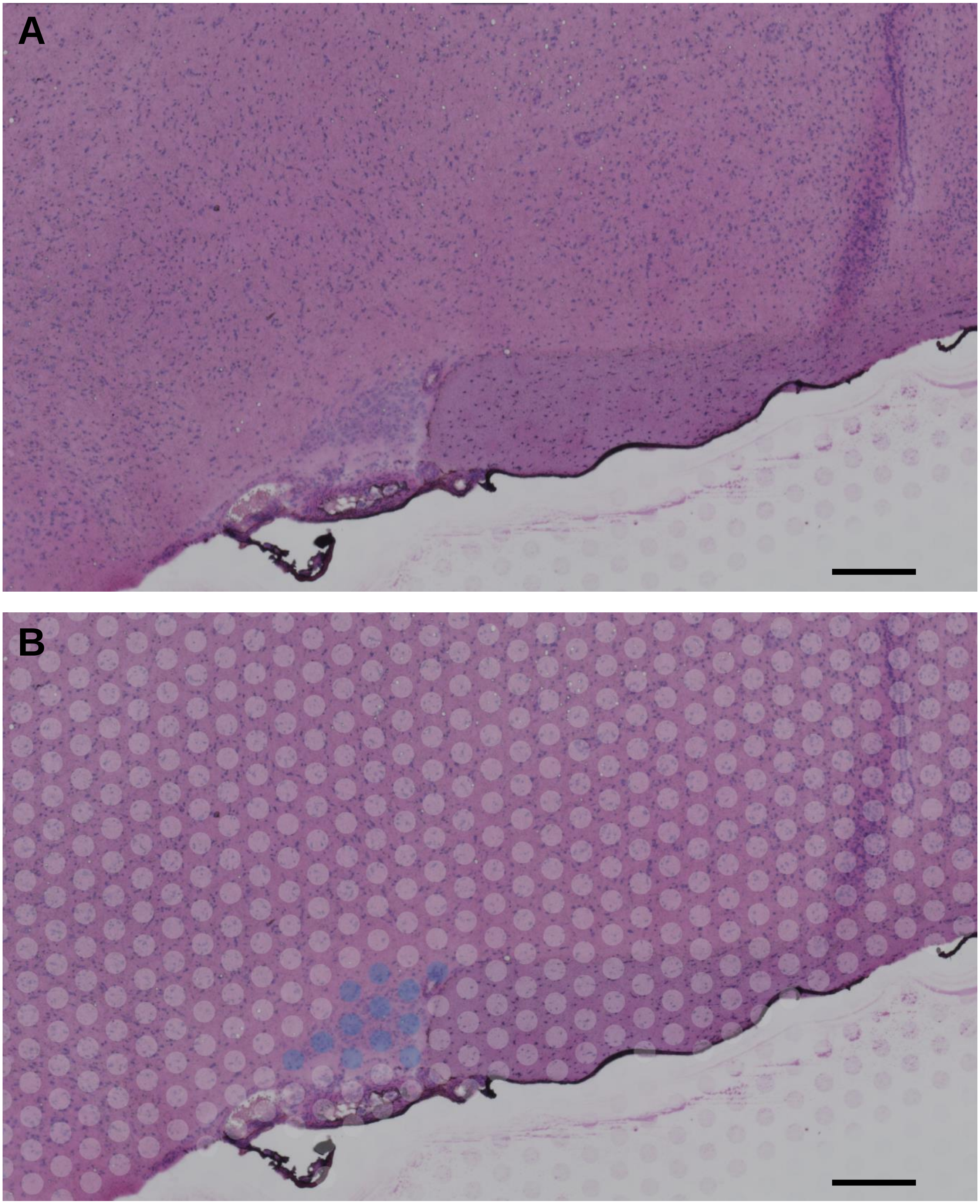

Supplemental Figure 6: Defining SON Region – Male 1. (A) Magnified H&E image of coronal brain section from Male 1. (B) Barcoded spots overlaying SON region of Male 1 selected (blue). Scale bar = 250 µm.

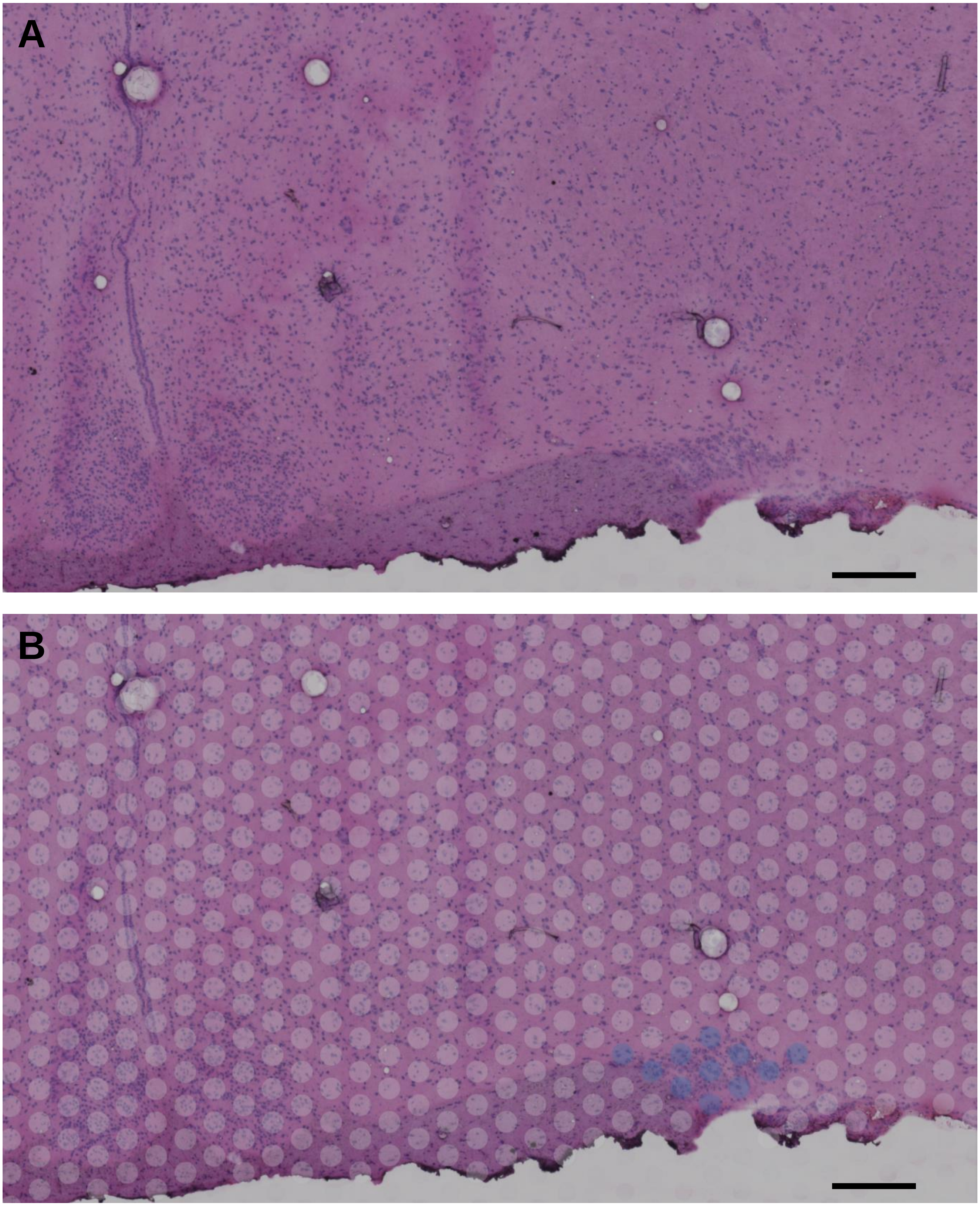

Supplemental Figure 7: Defining SON Region – Male 2. (A) Magnified H&E image of coronal brain section from Male 2. (B) Barcoded spots overlaying SON region of Male 2 selected (blue). Scale bar = 250 µm.

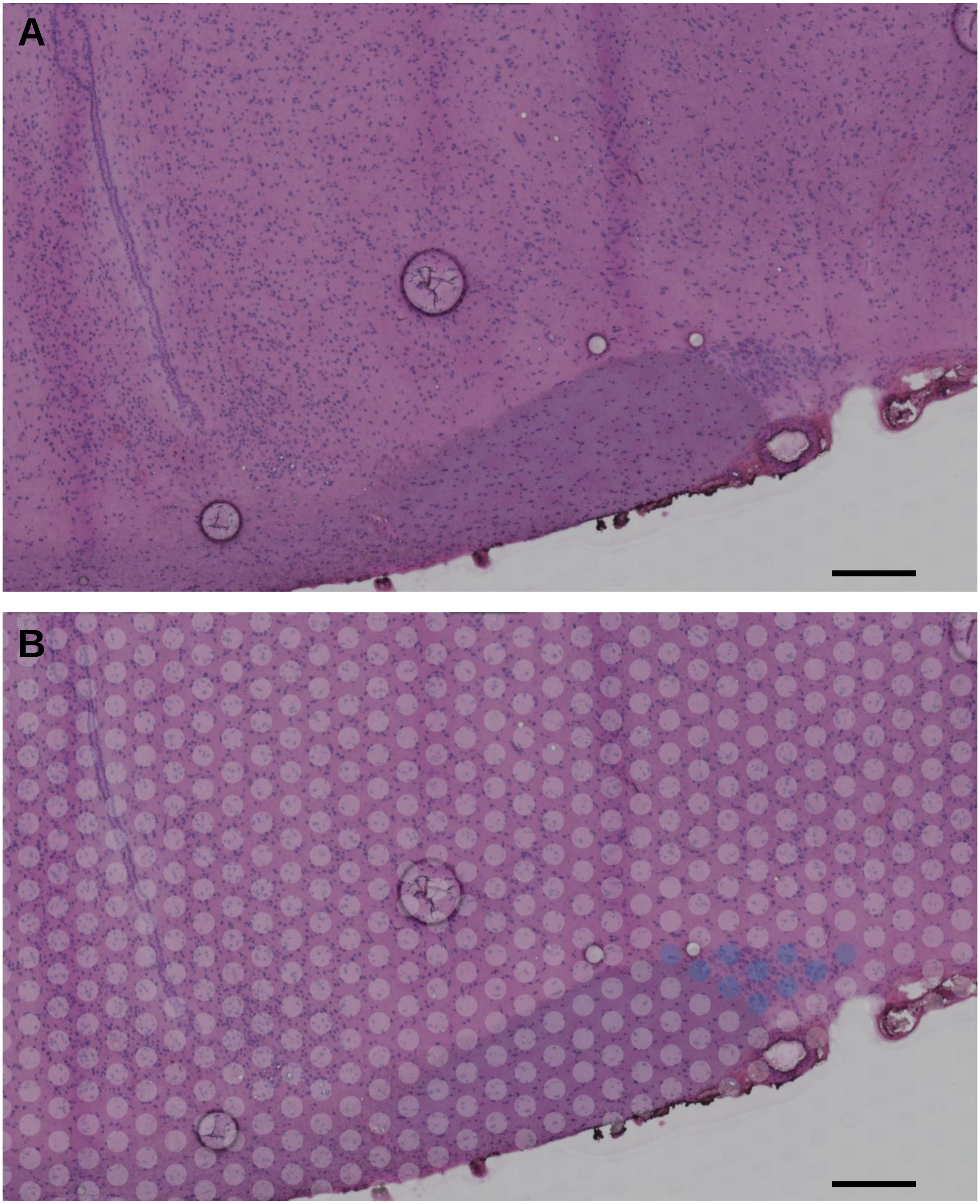

Supplemental Figure 8: Defining SON Region – Male 3. (A) Magnified H&E image of coronal brain section from Male 3. (B) Barcoded spots overlaying SON region of Male 3 selected (blue). Scale bar = 250 µm.

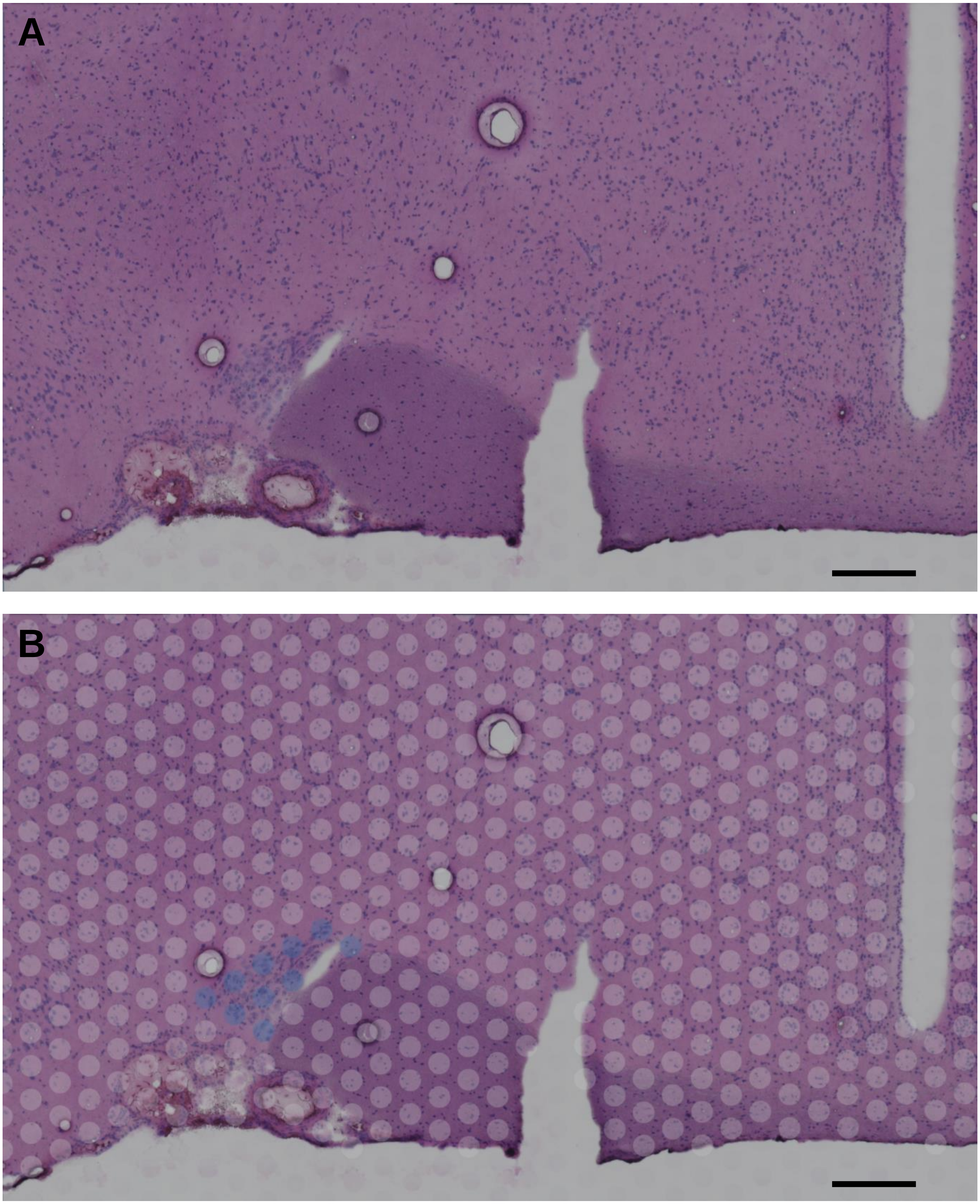

Supplemental Figure 9: Defining SON Region – Male 4. (A) Magnified H&E image of coronal brain section from Male 4. (B) Barcoded spots overlaying SON region of Male 4 selected (blue). Scale bar = 250 µm.

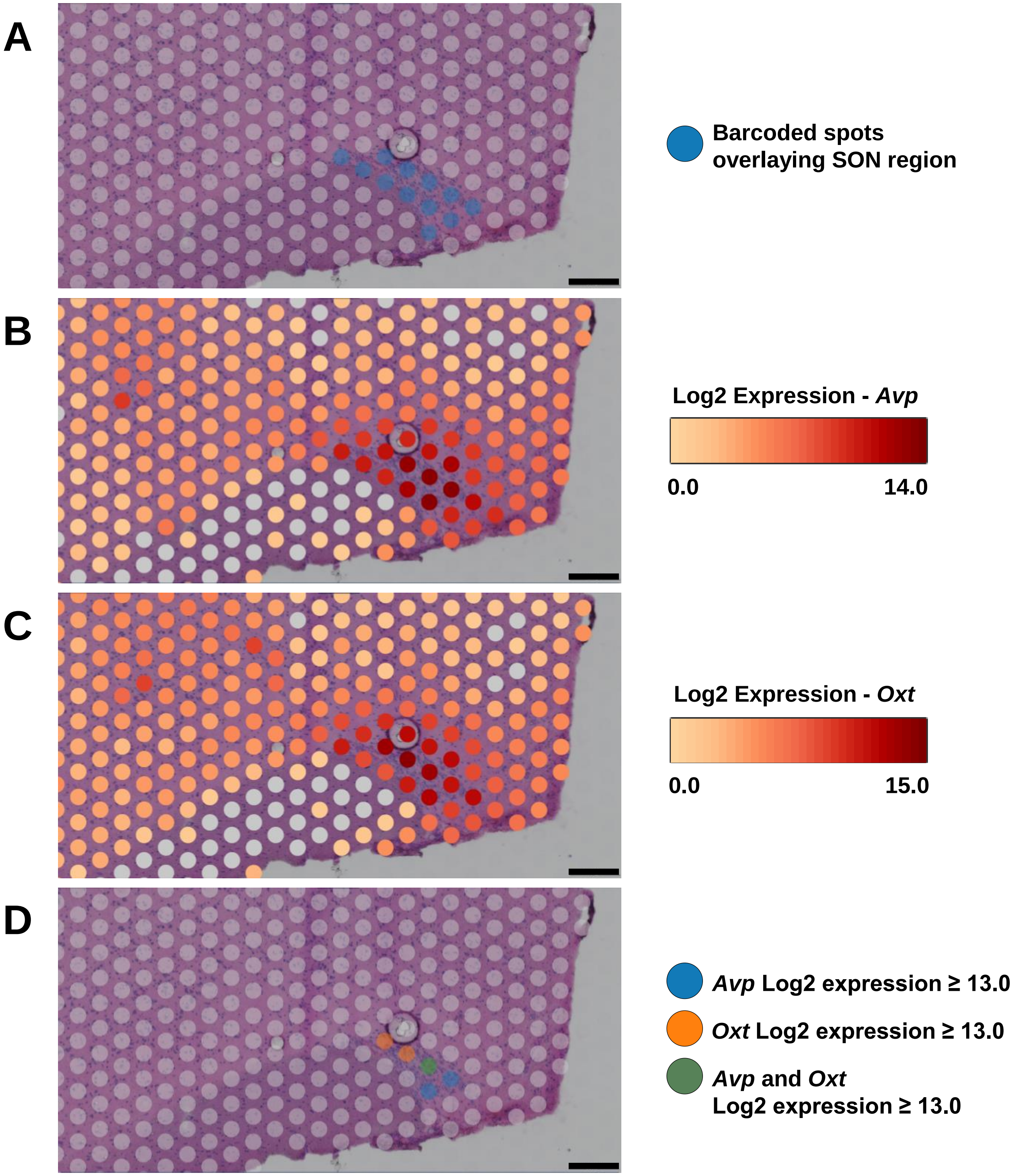

Supplemental Figure 10: Spatial Gene Expression Analysis for *Avp* and *Oxt* – Female 1. (A) Magnified H&E image of the coronal brain section in Supplemental Figure 2B from Female 1 showing barcoded spots overlaying the SON region (blue). For these barcoded spots, expression values were determined for *Avp* and *Oxt*, using a log2-transformed rendering of Unique Molecular Identifier (UMI) counts that represent the absolute number of observed transcripts. (B) A color-coded map of Log2 expression values for *Avp*, ranging from 0 (light peach) to 14 (dark red). (C) A color-coded map of Log2 expression values for *Oxt*, ranging from 0 (light peach) to 15 (dark red). Barcoded spots that highly expressed *Avp* and *Oxt* were identified in the SON. (D) Highest-expressing spots for *Avp* (blue), *Oxt* (yellow), and both *Avp* and *Oxt* (green) were identified by setting the Log2 expression threshold to ≥ 13.0. The predominantly *Avp*-expressing neurons tended to be located more in the ventral SON, while predominantly *Oxt*-expressing neurons were located more in the dorsal SON. This anatomical distribution of *Avp* and *Oxt* expression in the SON is consistent with the literature. Scale bar = 200 µm.

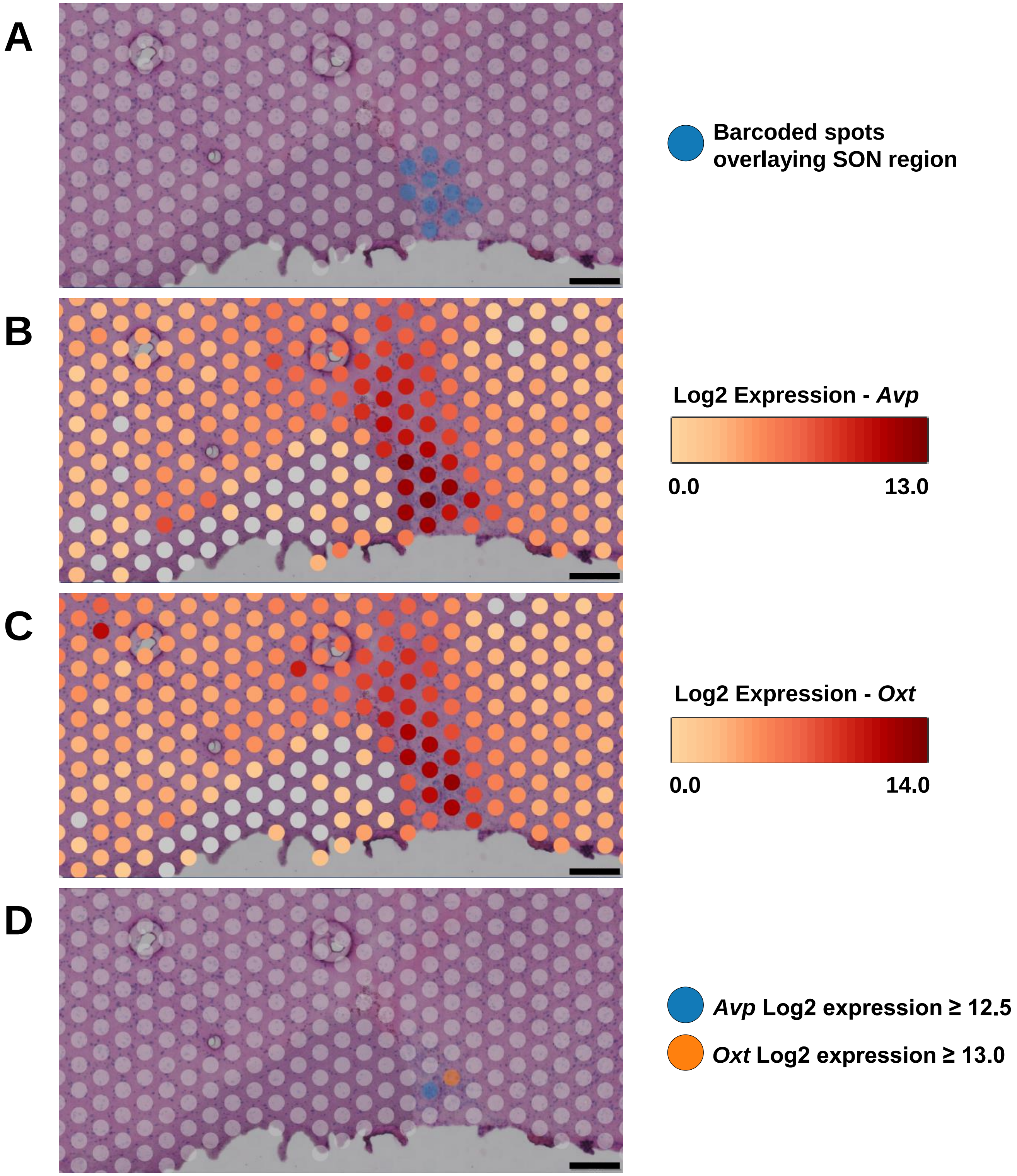

Supplemental Figure 11: Spatial Gene Expression Analysis for *Avp* and *Oxt* – Female 2. (A) Magnified H&E image of the coronal brain section in Supplemental Figure 3B from Female 2 showing barcoded spots overlaying the SON region (blue). The barcoded spots were used for determining *Avp* and *Oxt* expression in the SON as in the previous figure. B) A color-coded map of Log2 expression values for *Avp*, ranging from 0 (light peach) to 13 (dark red). (C) A color-coded map of Log2 expression values for *Oxt*, ranging from 0 (light peach) to 14 (dark red). (D) Highest-expressing spots for *Avp* (blue) and *Oxt* (yellow) were identified by setting the Log2 expression threshold to ≥ 12.5 for *Avp* and ≥ 13.0 for *Oxt*. Scale bar = 200 µm.

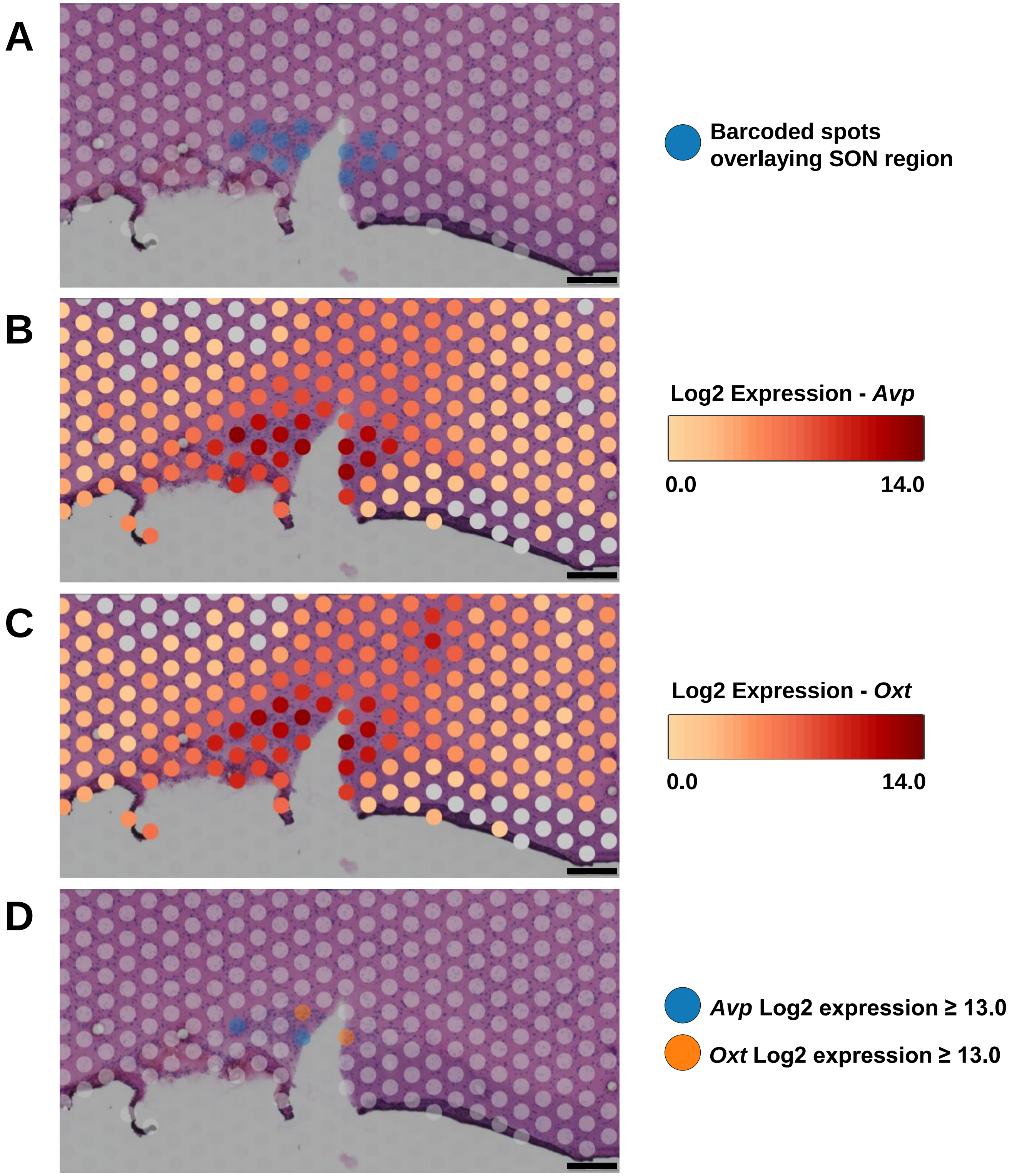

Supplemental Figure 12: Spatial Gene Expression Analysis for *Avp* and *Oxt* – Female 3. (A) Magnified H&E image of the coronal brain section in Supplemental Figure 4B from Female 3 showing barcoded spots overlaying the SON region (blue). The barcoded spots were used for determining *Avp* and *Oxt* expression in the SON as in the previous figure. B) A color-coded map of Log2 expression values for *Avp*, ranging from 0 (light peach) to 14 (dark red). (C) A color-coded map of Log2 expression values for *Oxt*, ranging from 0 (light peach) to 14 (dark red). (D) Highest-expressing spots for *Avp* (blue) and *Oxt* (yellow) were identified by setting the Log2 expression threshold to ≥ 13.0. Scale bar = 200 µm.

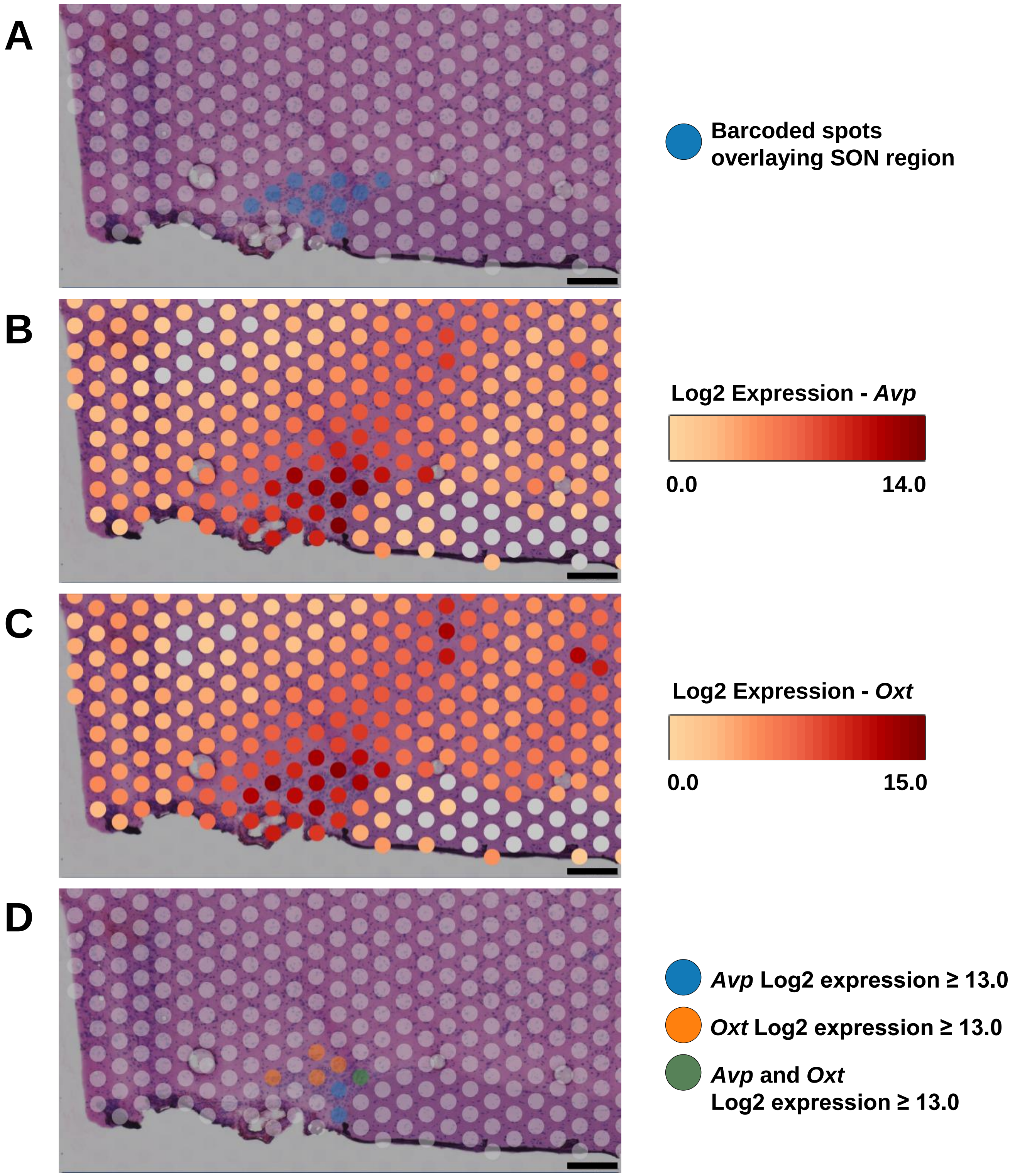

Supplemental Figure 13: Spatial Gene Expression Analysis for *Avp* and *Oxt* – Female 4. (A) Magnified H&E image of the coronal brain section in Supplemental Figure 5B from Female 4 showing barcoded spots overlaying the SON region (blue). The barcoded spots were used for determining *Avp* and *Oxt* expression in the SON as in the previous figure. B) A color-coded map of Log2 expression values for *Avp*, ranging from 0 (light peach) to 14 (dark red). (C) A color-coded map of Log2 expression values for *Oxt*, ranging from 0 (light peach) to 15 (dark red). (D) Highest-expressing spots for *Avp* (blue), *Oxt* (yellow), and both *Avp* and *Oxt* (green) were identified by setting the Log2 expression threshold to ≥ 13.0. Scale bar = 200 µm.

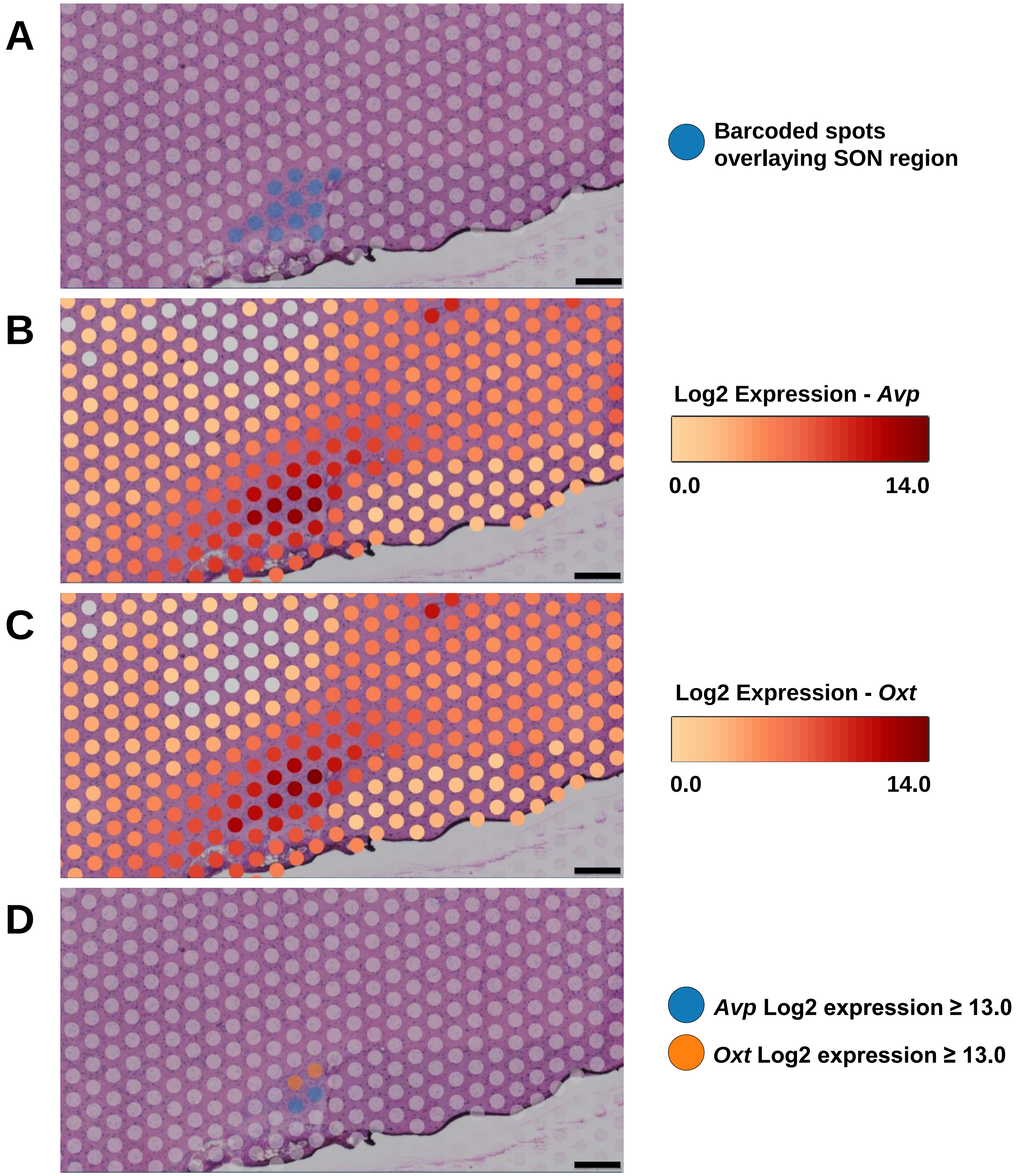

Supplemental Figure 14: Spatial Gene Expression Analysis for *Avp* and *Oxt* – Male 1. (A) Magnified H&E image of the coronal brain section in Supplemental Figure 6B from Male 1 showing barcoded spots overlaying the SON region (blue). The barcoded spots were used for determining *Avp* and *Oxt* expression in the SON as in the previous figure. B) A color-coded map of Log2 expression values for *Avp*, ranging from 0 (light peach) to 14 (dark red). (C) A color-coded map of Log2 expression values for *Oxt*, ranging from 0 (light peach) to 14 (dark red). (D) Highest-expressing spots for *Avp* (blue) and *Oxt* (yellow) were identified by setting the Log2 expression threshold to ≥ 13.0. Scale bar = 200 µm.

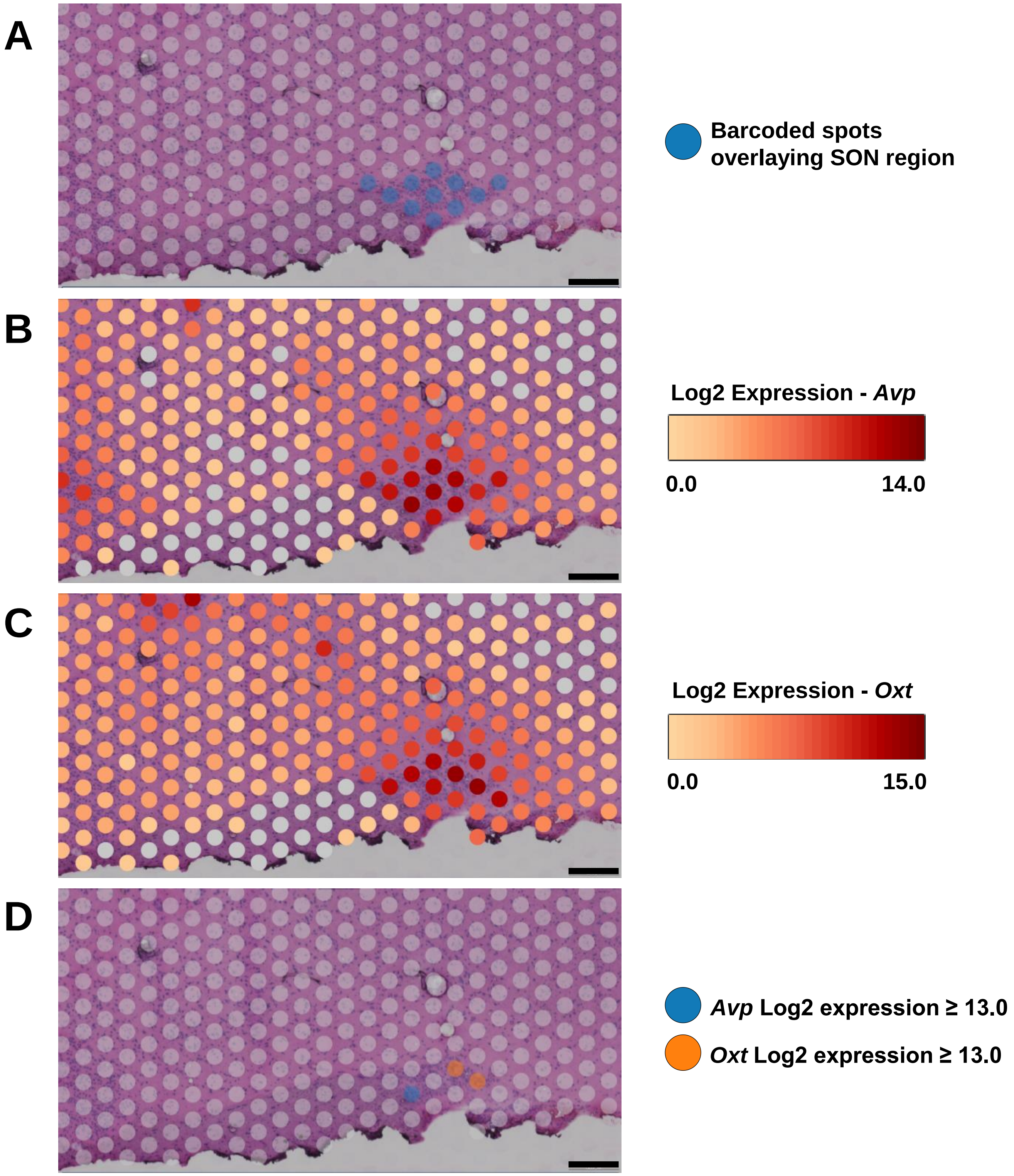

Supplemental Figure 15: Spatial Gene Expression Analysis for *Avp* and *Oxt* – Male 2. (A) Magnified H&E image of the coronal brain section in Supplemental Figure 7B from Male 2 showing barcoded spots overlaying the SON region (blue). The barcoded spots were used for determining *Avp* and *Oxt* expression in the SON as in the previous figure. B) A color-coded map of Log2 expression values for *Avp*, ranging from 0 (light peach) to 14 (dark red). (C) A color-coded map of Log2 expression values for *Oxt*, ranging from 0 (light peach) to 15 (dark red). (D) Highest-expressing spots for *Avp* (blue) and *Oxt* (yellow) were identified by setting the Log2 expression threshold to ≥ 13.0. Scale bar = 200 µm.

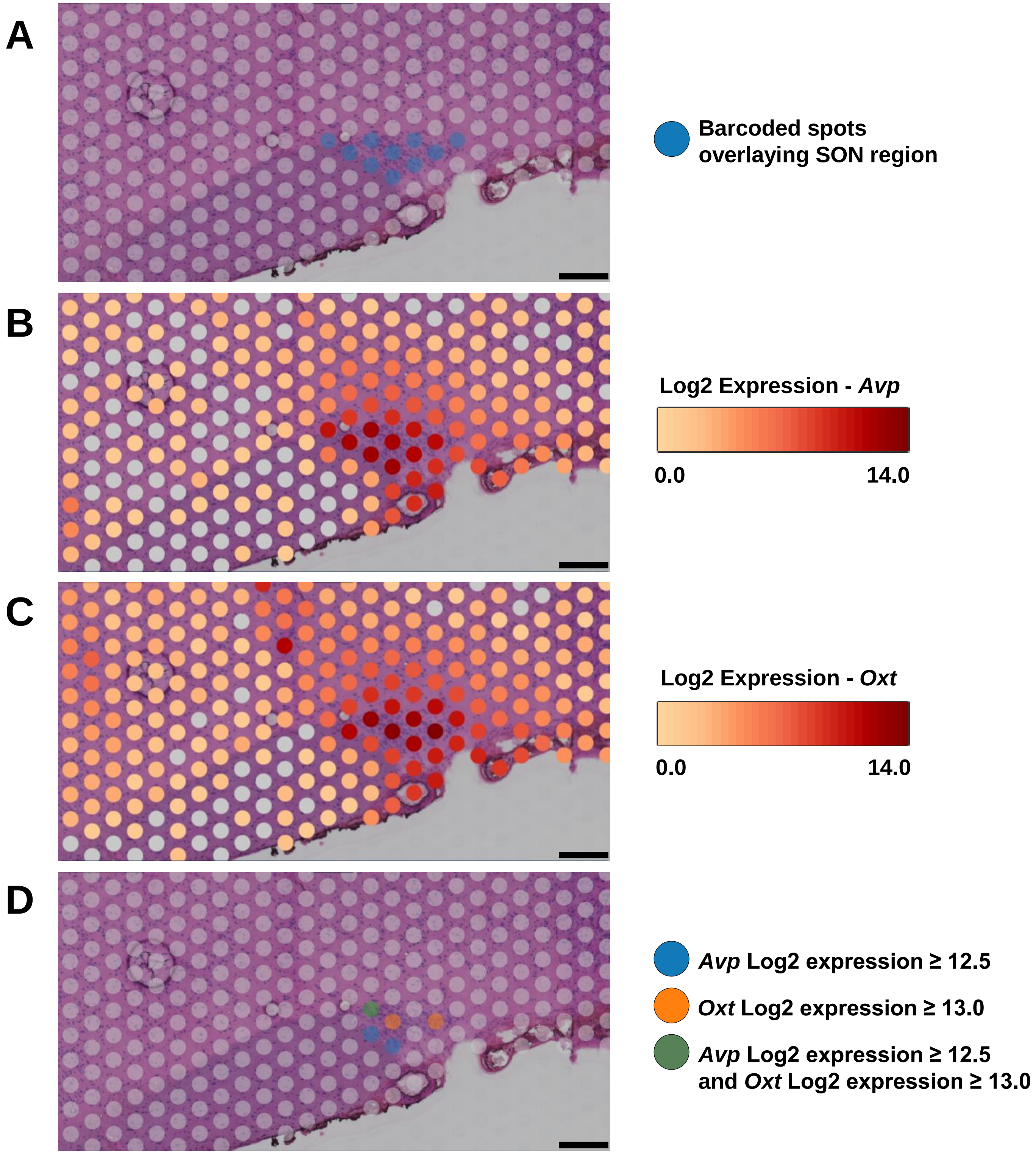

Supplemental Figure 16: Spatial Gene Expression Analysis for *Avp* and *Oxt* – Male 3. (A) Magnified H&E image of the coronal brain section in Supplemental Figure 8B from Male 3 showing barcoded spots overlaying the SON region (blue). The barcoded spots were used for determining *Avp* and *Oxt* expression in the SON as in the previous figure. B) A color-coded map of Log2 expression values for *Avp*, ranging from 0 (light peach) to 14 (dark red). (C) A color-coded map of Log2 expression values for *Oxt*, ranging from 0 (light peach) to 14 (dark red). (D) Highest-expressing spots for *Avp* (blue), *Oxt* (yellow), and both *Avp* and *Oxt* (green) were identified by setting the Log2 expression threshold to ≥ 12.5 for *Avp* and ≥ 13.0 for *Oxt*. Scale bar = 200 µm.

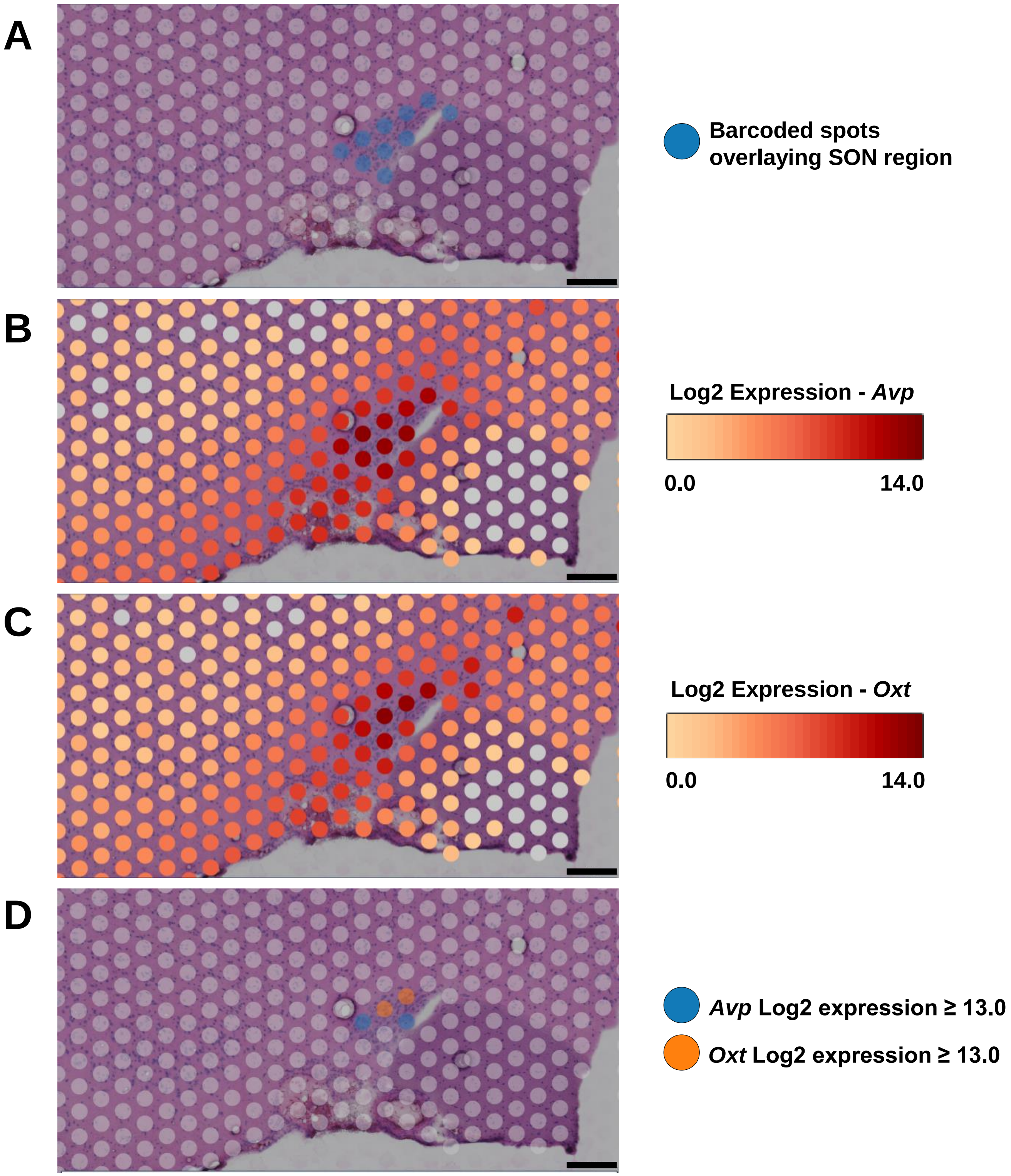

Supplemental Figure 17: Spatial Gene Expression Analysis for *Avp* and *Oxt* – Male 4. (A) Magnified H&E image of the coronal brain section in Supplemental Figure 9B from Male 4 showing barcoded spots overlaying the SON region (blue). The barcoded spots were used for determining *Avp* and *Oxt* expression in the SON as in the previous figure. B) A color-coded map of Log2 expression values for *Avp*, ranging from 0 (light peach) to 14 (dark red). (C) A color-coded map of Log2 expression values for *Oxt*, ranging from 0 (light peach) to 14 (dark red). (D) Highest-expressing spots for *Avp* (blue) and *Oxt* (yellow) were identified by setting the Log2 expression threshold to ≥ 13.0. Scale bar = 200 μm.

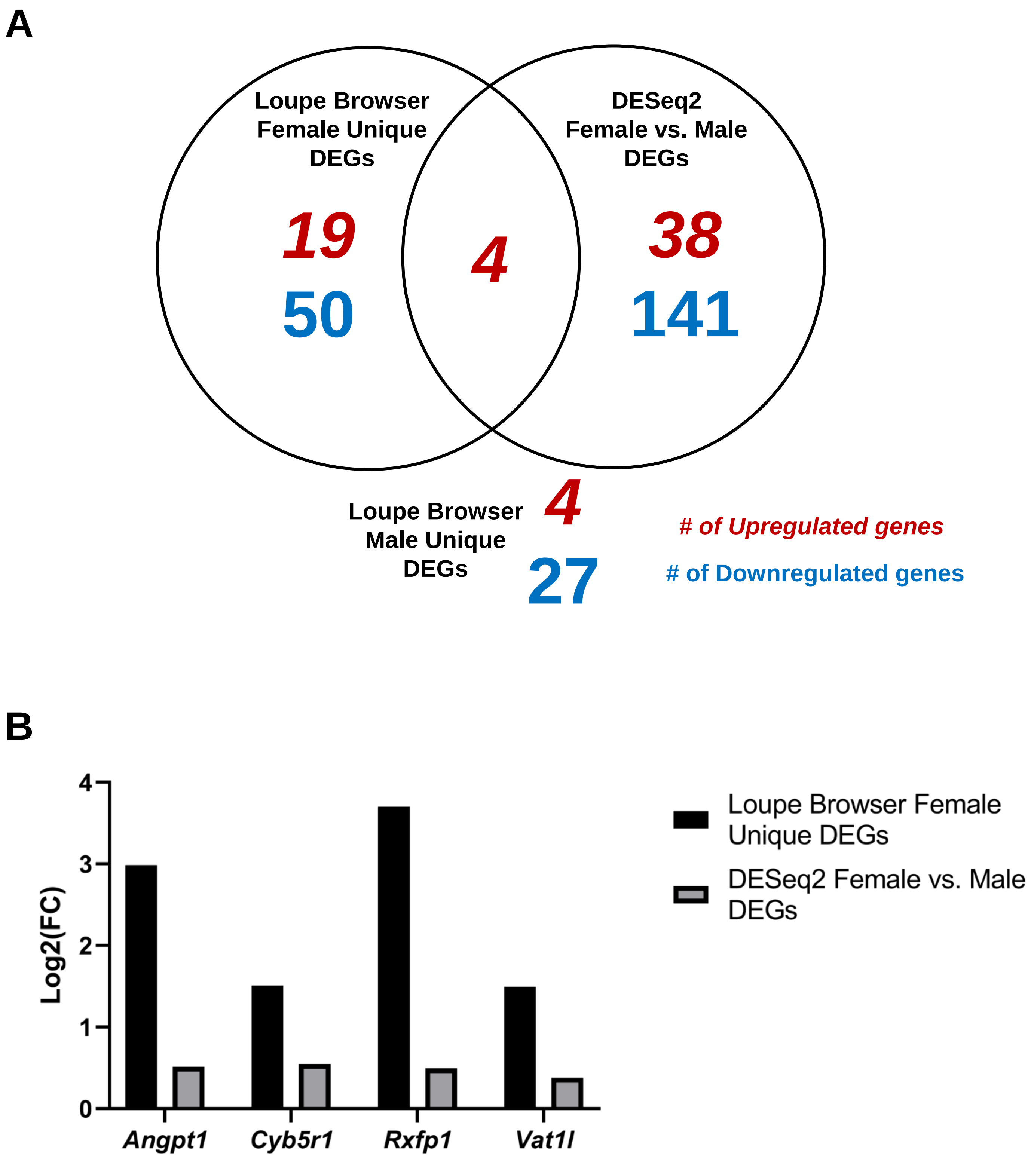

Supplemental Figure 18: Comparison of Loupe Browser DEGs Unique to Female and Male Groups to DESeq2 Female vs. Male DEGs. (A) Venn diagram displaying the number of upregulated (red) and downregulated (blue) DEGs unique to the female and male groups identified by Loupe Browser and DESeq2 Female vs. Male DEGs. Four genes were found to be common between Loupe Browser Female Unique DEGs and DESeq2 Female vs. Male DEGs. (B) Bar graph of Log2 fold change values for the four common DEGs.

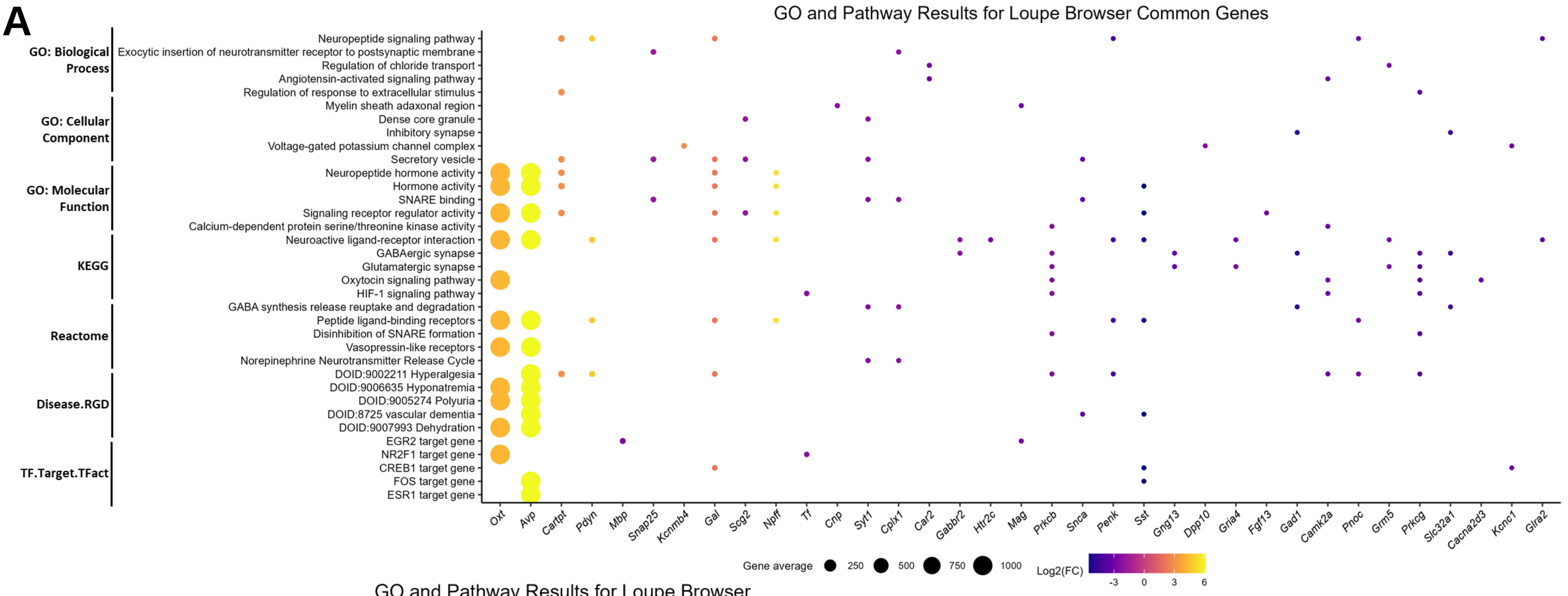

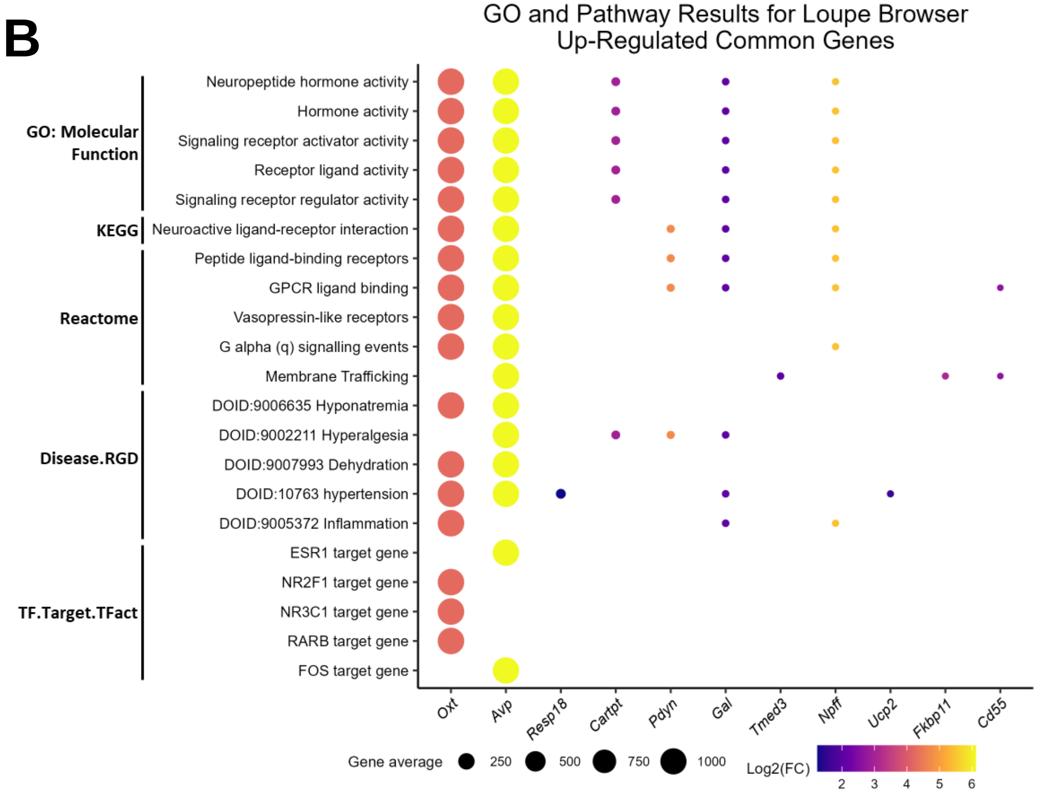

Supplemental Figure 19: Top 5 GO Terms/Pathways per Category using Loupe Browser Common SON DEGs. The dot plots highlight up to top 5 GO term/pathway results per category based on most significant enrichment FDR for Loupe Browser Common SON DEGs (A) and when only upregulated SON DEGs common to both sexes were used as data input (B). KEGG = Kyoto Encyclopedia of Genes and Genomes; RGD = Rat Genome Database; Disease RGD = RGD disease portals; TF = Transcription Factor from TF.Target.TFact database. Dot size = gene average; color scale = Log2 fold change; females (n= 4); males (n=4).

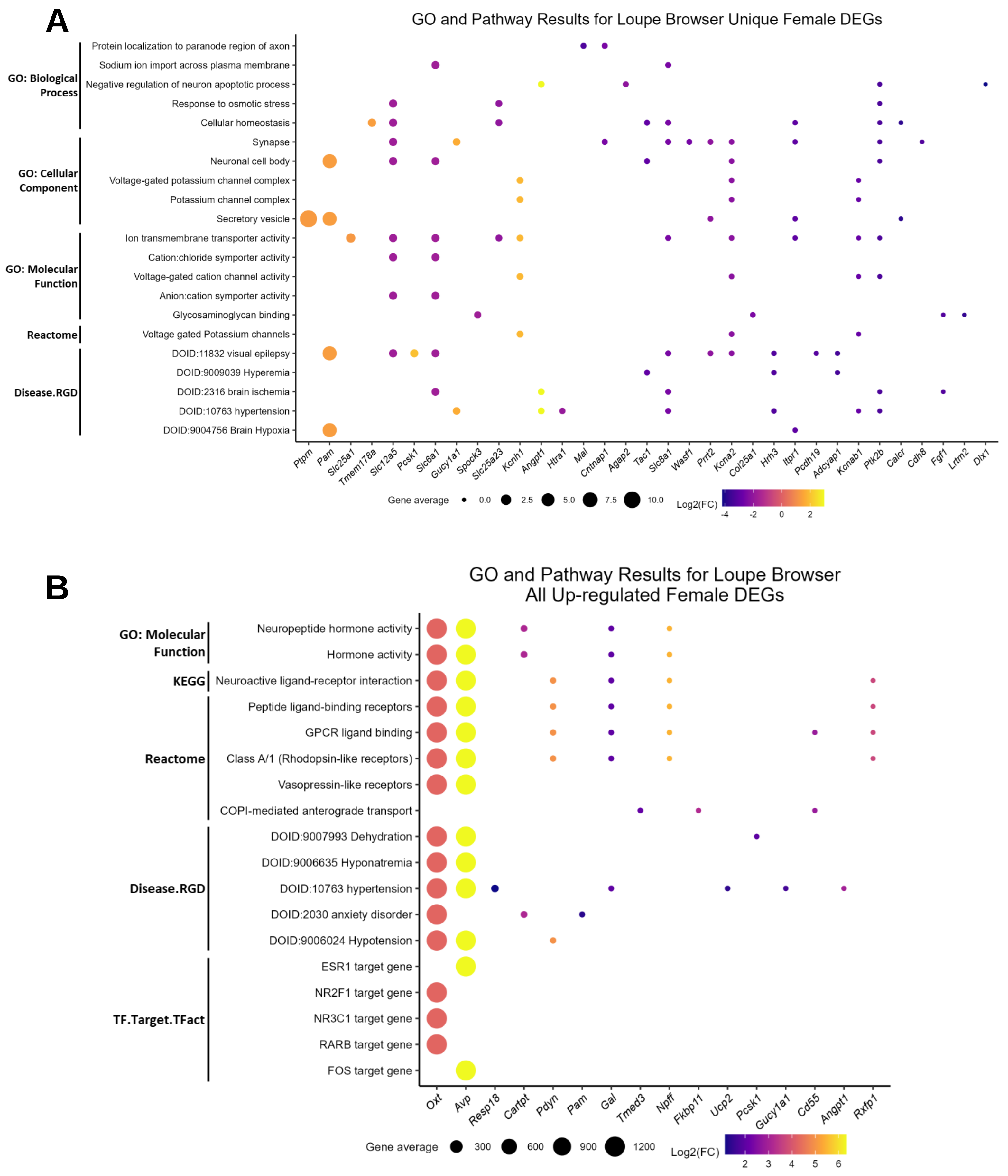

Supplemental Figure 20: Top 5 GO Terms/Pathways per Category using Loupe Browser Female SON DEGs. The dot plots highlight up to top 5 GO term/pathway results per category based on most significant enrichment FDR for Loupe Browser female-unique SON DEGs (A) and when all upregulated female SON DEGs were used as data input (B). KEGG = Kyoto Encyclopedia of Genes and Genomes; RGD = Rat Genome Database; Disease RGD = RGD disease portals; TF = Transcription Factor from TF.Target.TFact database. Dot size = gene average; color scale = Log2 fold change; females (n= 4).

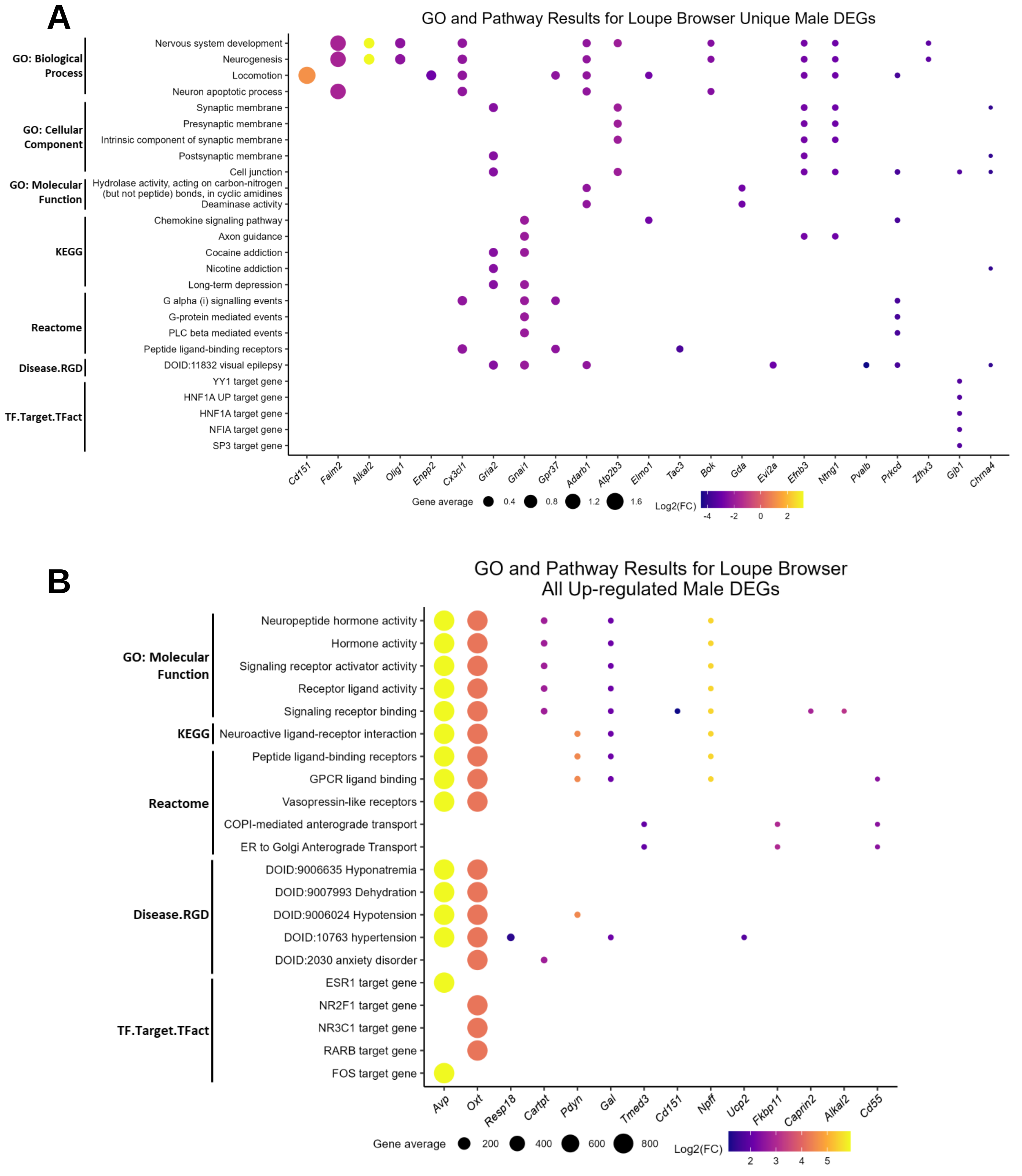

Supplemental Figure 21: Top 5 GO Terms/Pathways per Category using Loupe Browser Male SON DEGs. The dot plots highlight up to top 5 GO term/pathway results per category based on most significant enrichment FDR for Loupe Browser male-unique SON DEGs (A) and when all upregulated male SON DEGs were used as data input (B). KEGG = Kyoto Encyclopedia of Genes and Genomes; RGD = Rat Genome Database; Disease RGD = RGD disease portals; TF = Transcription Factor from TF.Target.TFact database. Dot size = gene average; color scale = Log2 fold change; males (n= 4).

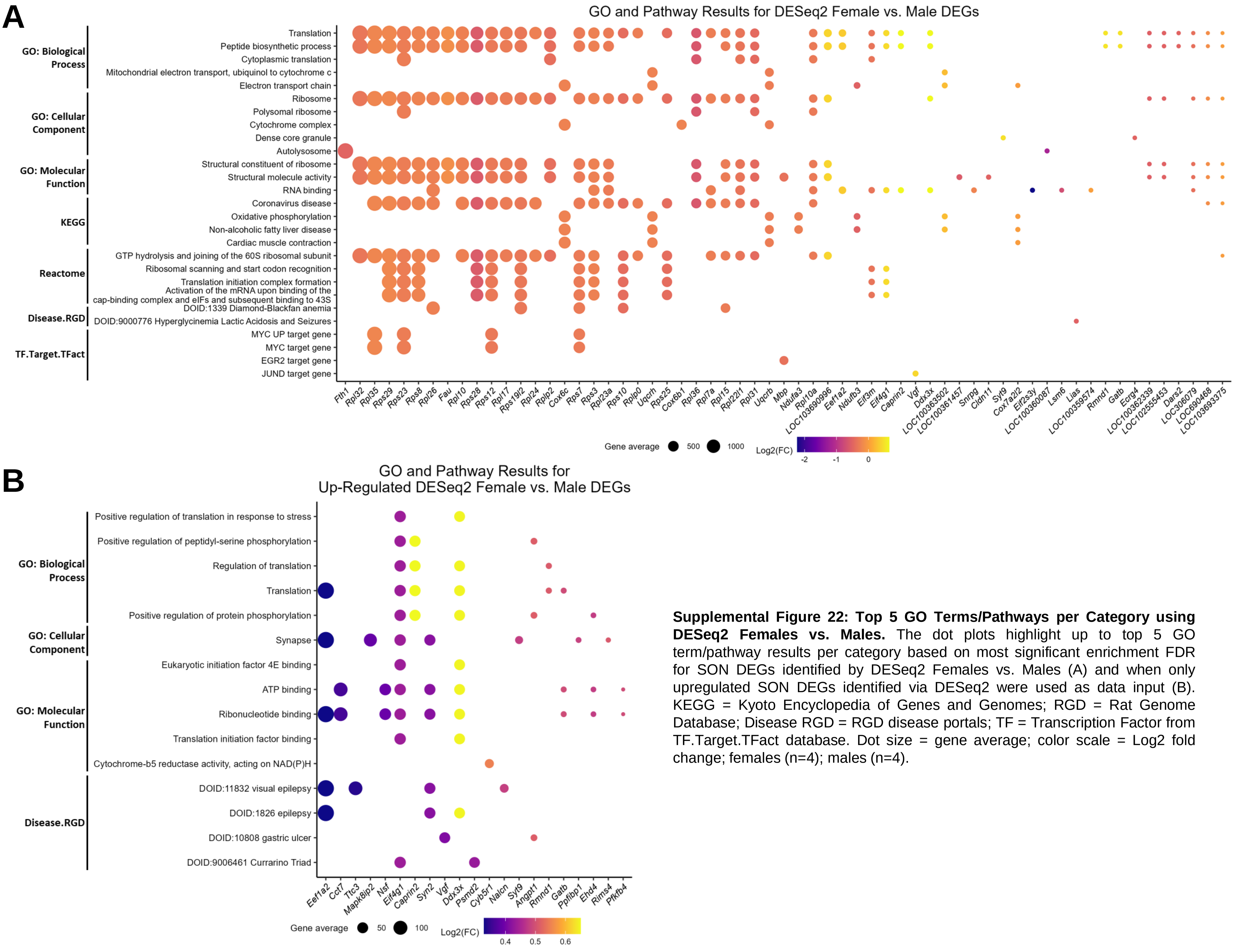

Supplemental Figure 22: Top 5 GO Terms/Pathways per Category using DESeq2 Females vs. Males. The dot plots highlight up to top 5 GO term/pathway results per category based on most significant enrichment FDR for SON DEGs identified by DESeq2 Females vs. Males (A) and when only upregulated SON DEGs identified via DESeq2 were used as data input (B). KEGG = Kyoto Encyclopedia of Genes and Genomes; RGD = Rat Genome Database; Disease RGD = RGD disease portals; TF = Transcription Factor from TF.Target.TFact database. Dot size = gene average; color scale = Log2 fold change; females (n=4); males (n=4).

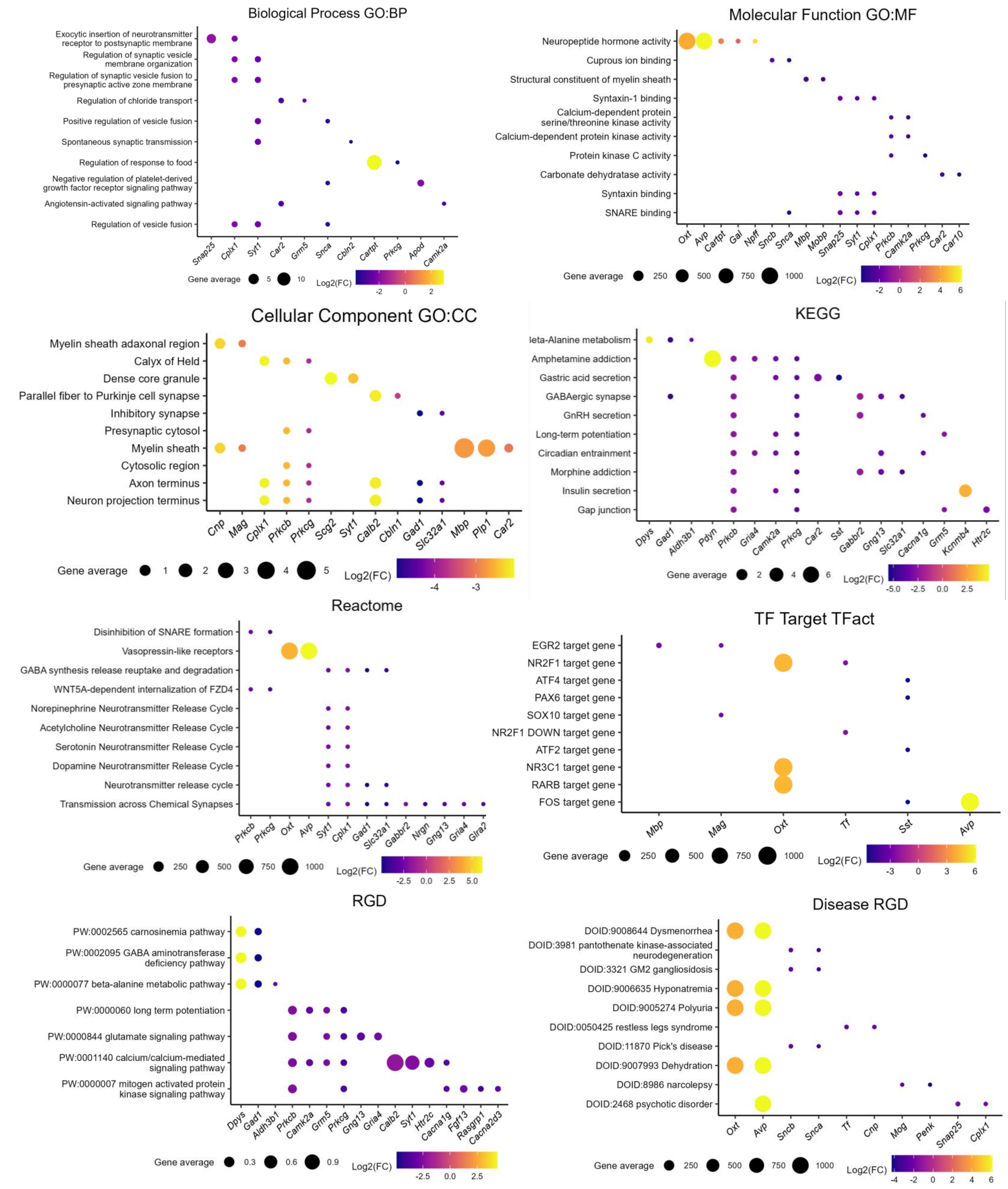

Supplemental Figure 23: Top 10 GO Terms/Pathways per Category using Loupe Browser Common Genes. The dot plots highlight top 10 GO term/pathway results per category based on fold enrichment. KEGG = Kyoto Encyclopedia of Genes and Genomes; RGD = Rat Genome Database; Disease RGD = RGD disease portals; TF = Transcription Factor from TF.Target.TFact database. Dot size = gene average; color scale = Log2 fold change; n= 8.

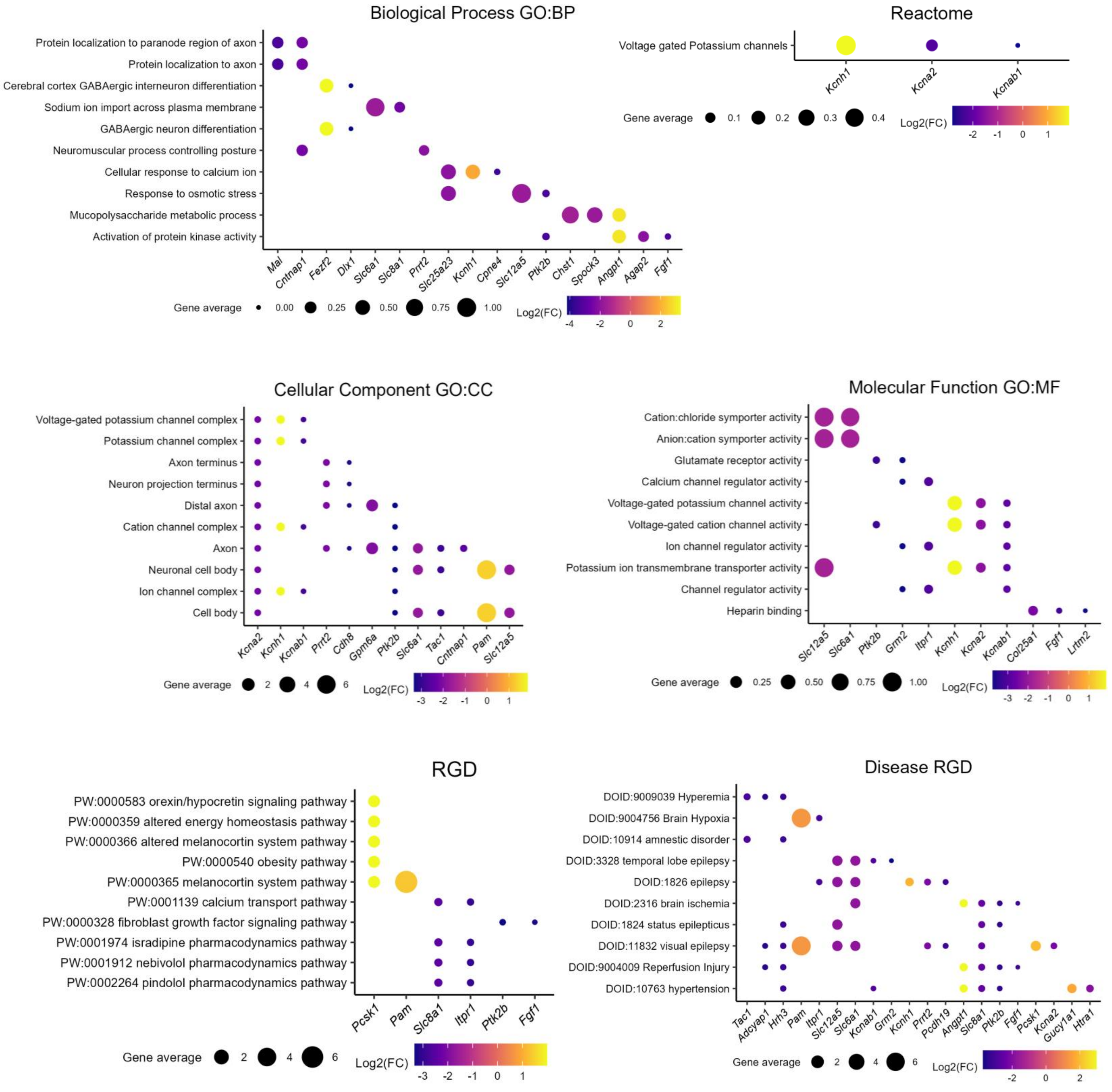

Supplemental Figure 24: Top 10 GO Terms/Pathways per Category using Loupe Browser Female-Unique Genes. The dot plots highlight top 10 GO term/pathway results per category based on fold enrichment. RGD = Rat Genome Database; Disease RGD = RGD disease portals. Dot size = gene average; color scale = Log2 fold change; n= 4.

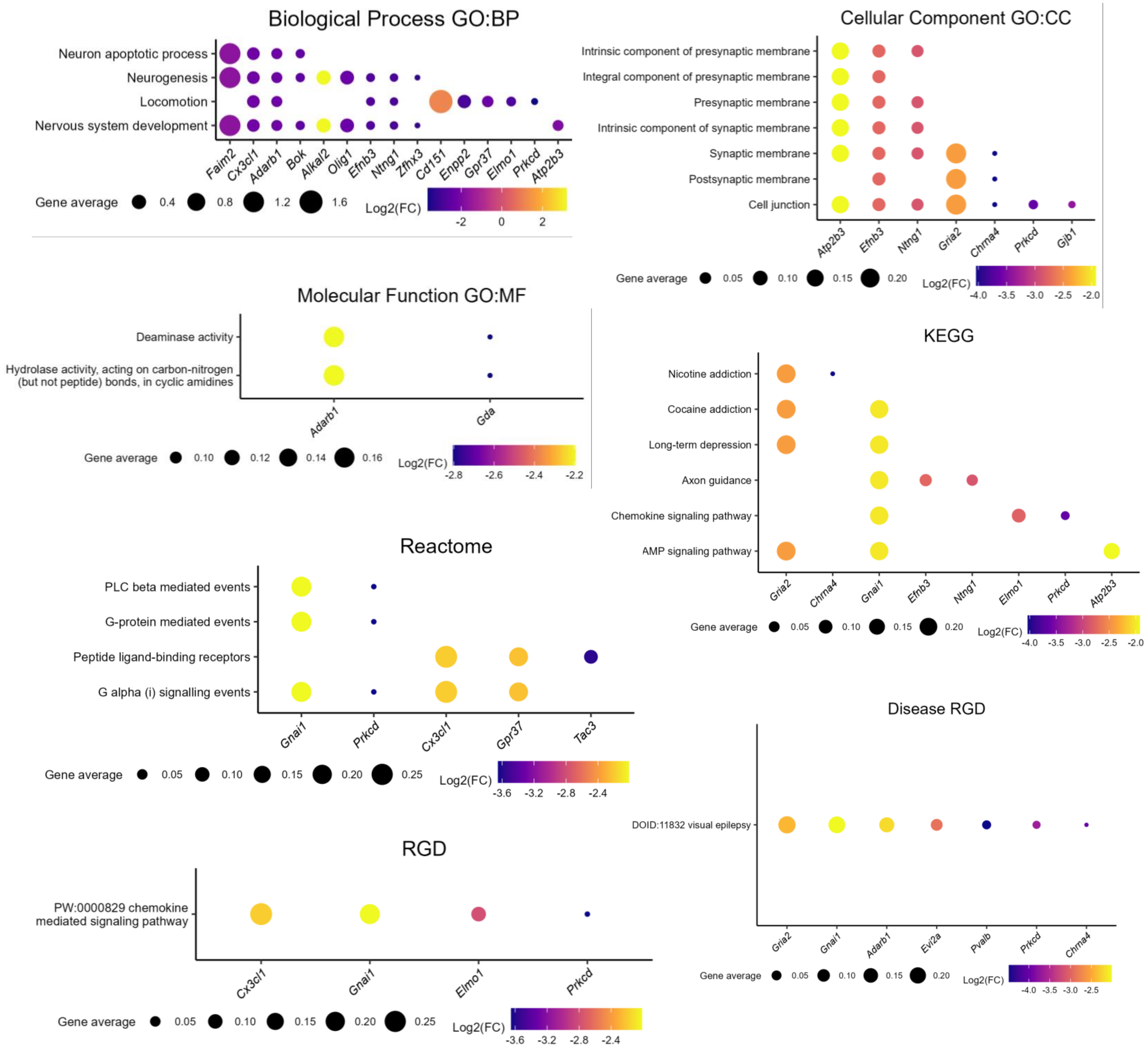

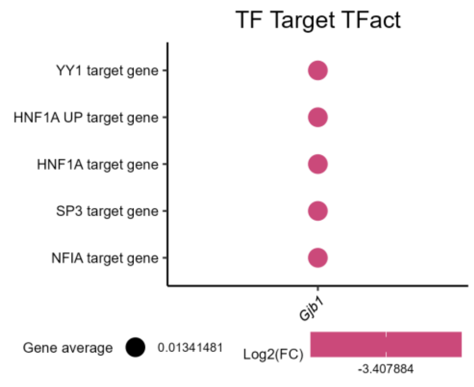

Supplemental Figure 25: Top 10 GO Terms/Pathways per Category using Loupe Browser Male-Unique Genes. The dot plots highlight top 10 GO term/pathway results per category based on fold enrichment. KEGG = Kyoto Encyclopedia of Genes and Genomes; RGD = Rat Genome Database; Disease RGD = RGD disease portals; TF = Transcription Factor from TF.Target.TFact database. Dot size = gene average; color scale = Log2 fold change; n= 4.

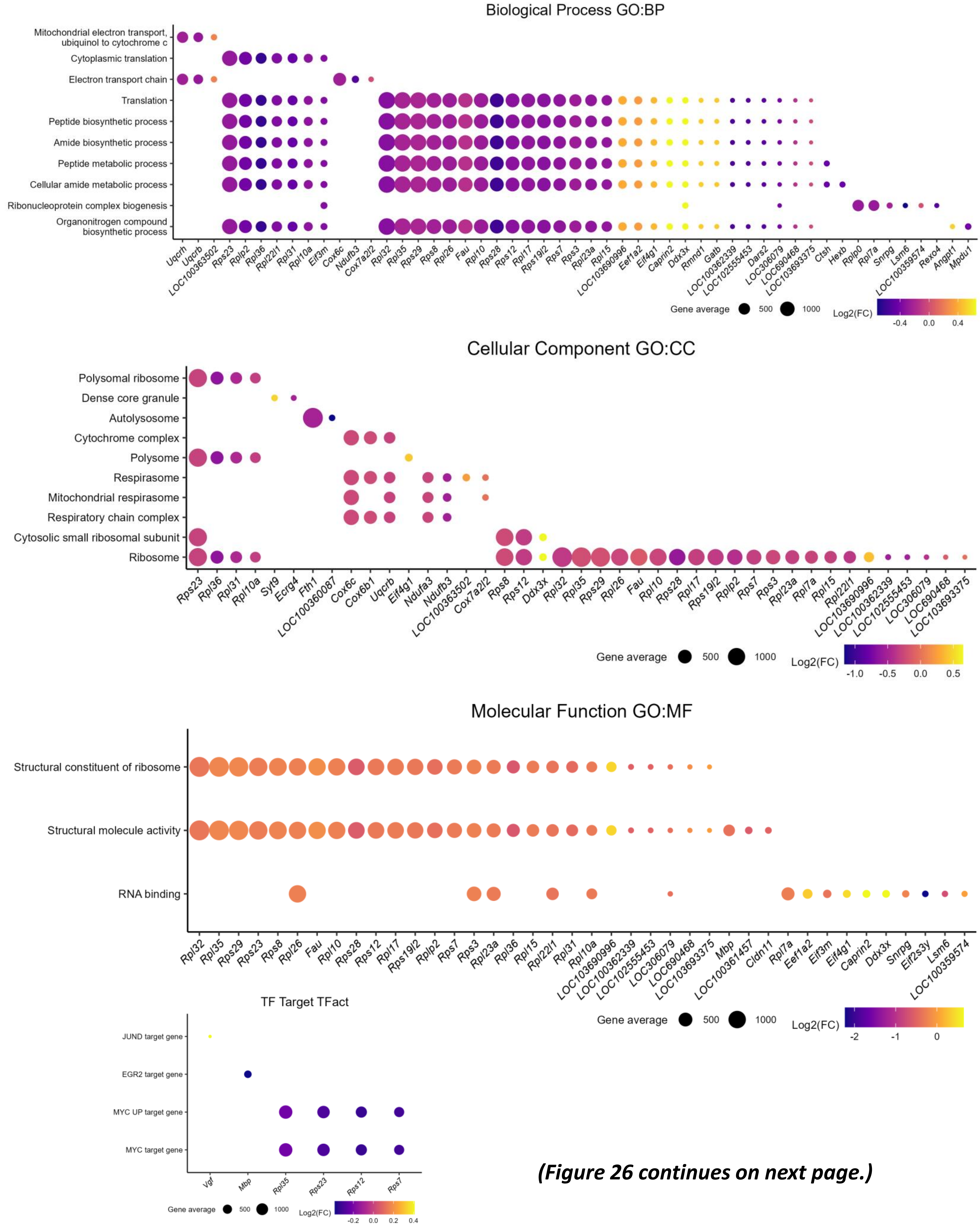

(Figure 26 continues on next page.)

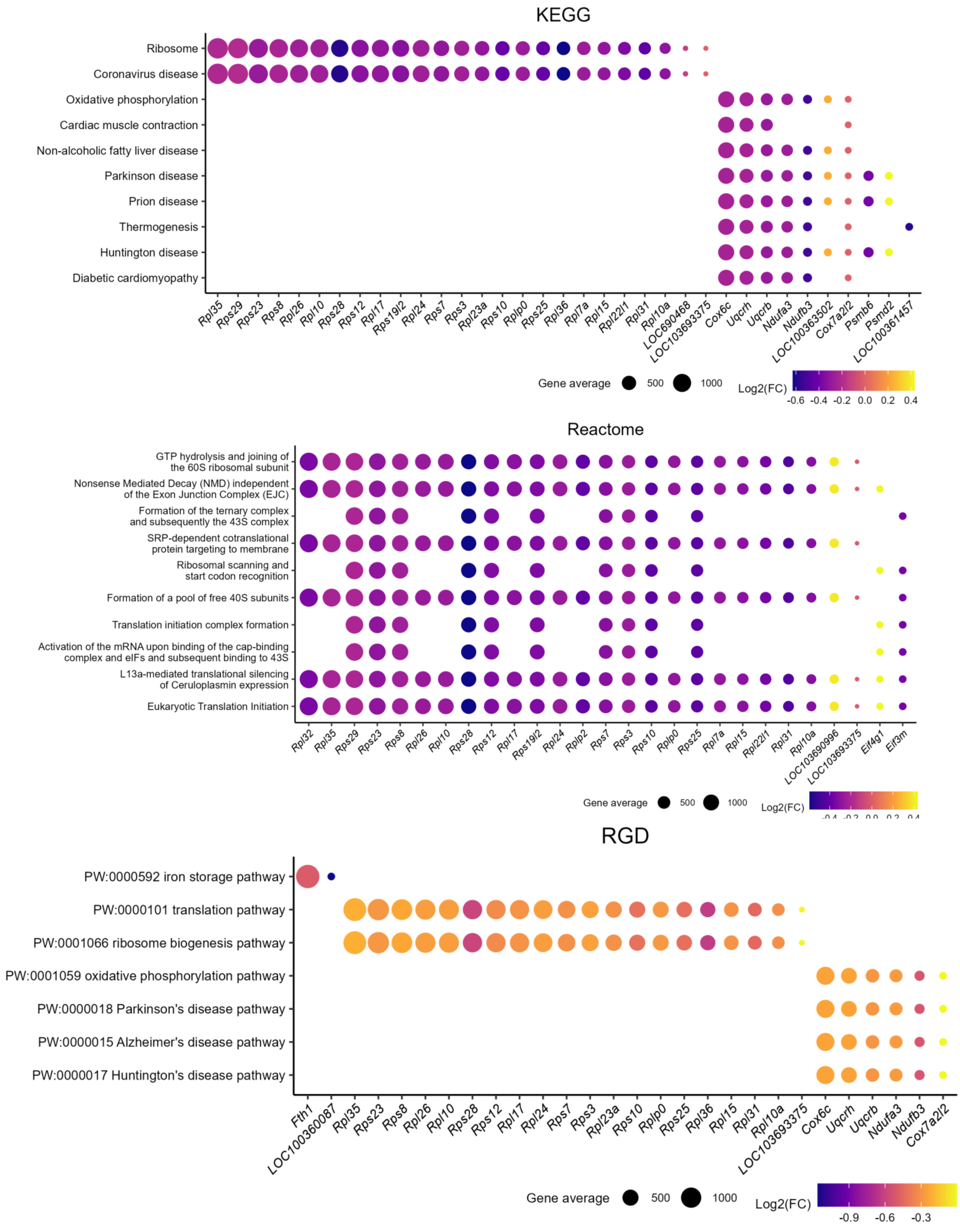

Supplemental Figure 26: Top 10 GO Terms/Pathways per Category using DESeq2 Females vs. Males. The dot plots highlight top 10 GO term/pathway results per category based on fold enrichment. KEGG = Kyoto Encyclopedia of Genes and Genomes; RGD = Rat Genome Database; Disease RGD = RGD disease portals; TF = Transcription Factor from TF.Target.TFact database. Dot size = gene average; color scale = Log2 fold change; females (n=4); males (n=4).

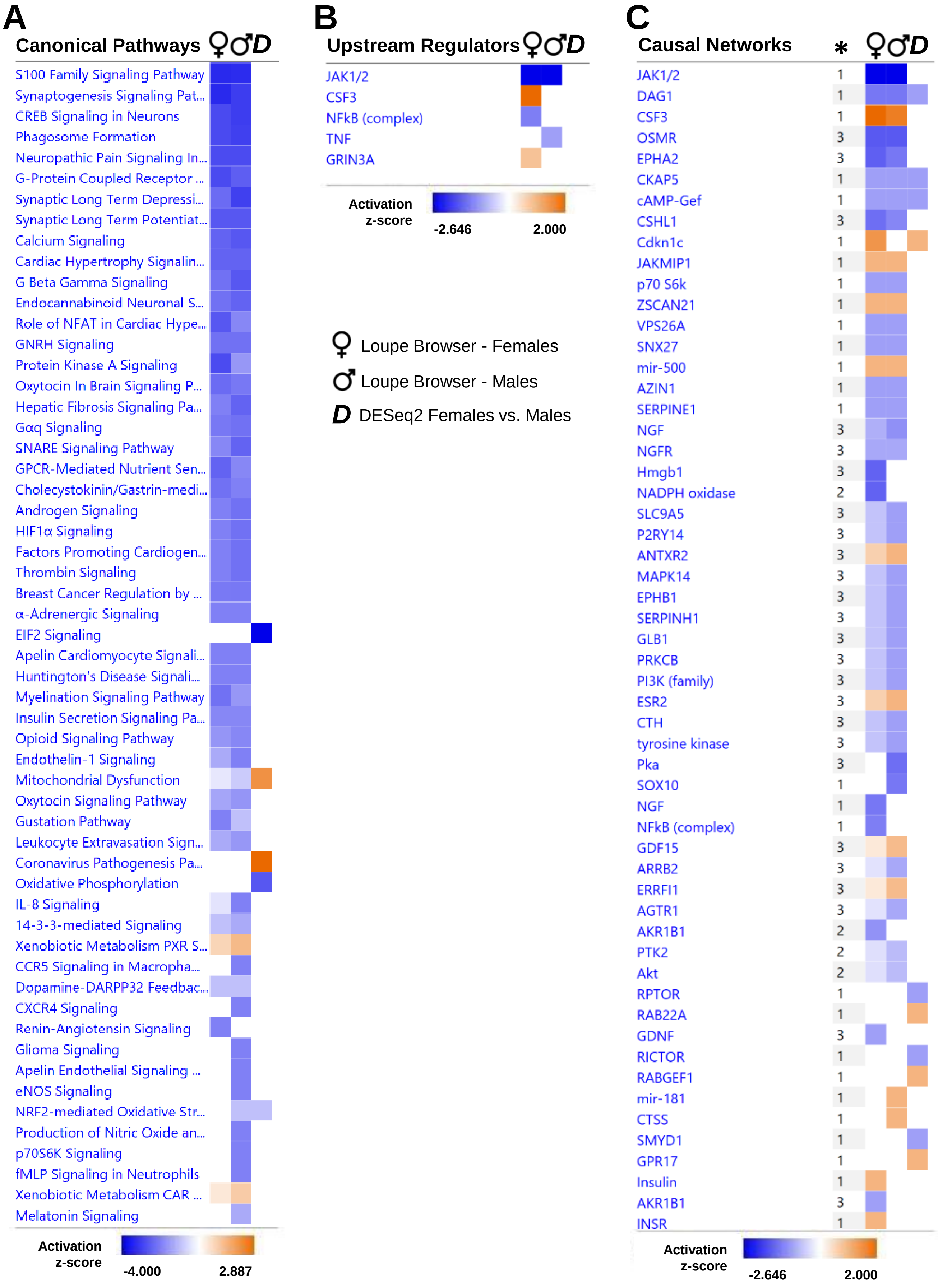

(Figure 27 continues on next page.)

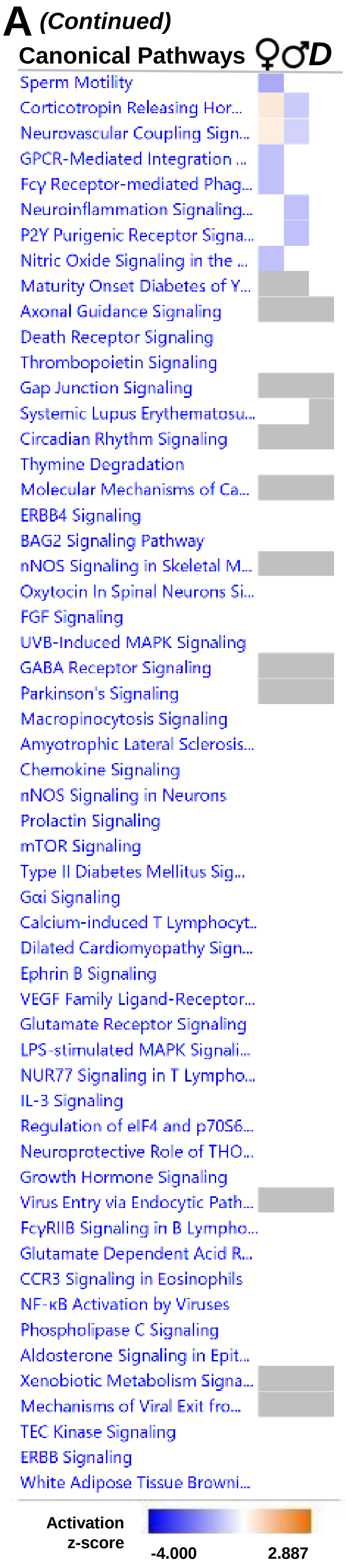

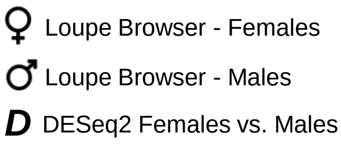

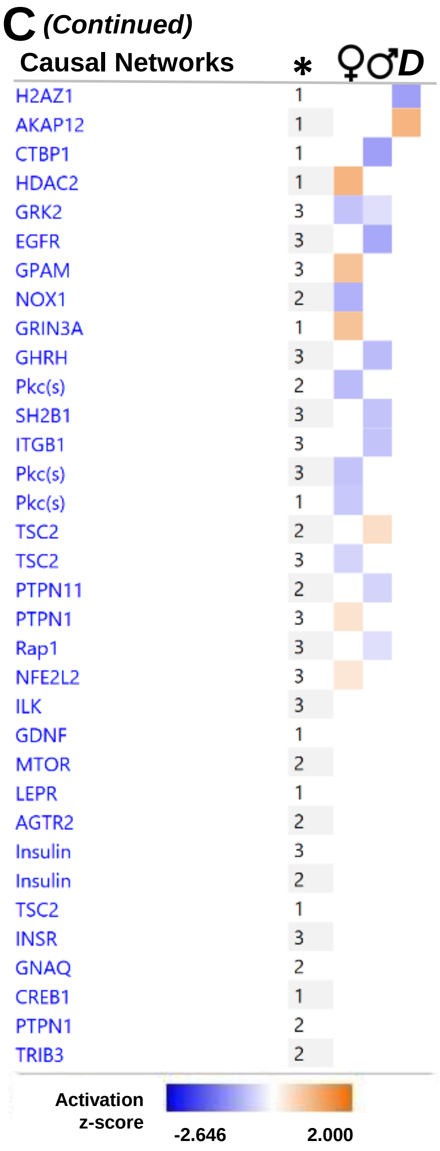

Supplemental Figure 27: Expanded IPA Comparison Analysis Results. Full list of IPA comparisons expanding on results presented in Figure 8 on canonical pathways (A), upstream regulators (B), and causal networks (C) identified for significant DEGs from DESeq2 (female vs. male group), LB Female Group (n=4), and LB Male Group (n=4). * indicates causal network depth according to the degree of separation between the upstream regulator and downstream target molecules in the data set.
